# Unraveling the diversity, function, and virus–host interactions of archaeal proviruses

**DOI:** 10.1093/ismeco/ycag243

**Published:** 2026-08-22

**Authors:** Yang Zhao, PengFei Liu, LinXing Chen, MeiLing Feng, Rong Wen, ZhiHao Zhang, XueFeng Zhang, HongFei Chi

**Affiliations:** Center for Pan-third Pole Environment, Lanzhou University, Lanzhou, 730000, China; Key Laboratory of Pan-third Pole Biogeochemical Cycling, Lanzhou University, Lanzhou, 730000, China; Chayu Monsoon Corridor Observation and Research Station for Multi-Sphere Changes, Lanzhou University, Chayu, 860600, China; College of Ecology, Lanzhou University, Lanzhou, 730000, China; Center for Pan-third Pole Environment, Lanzhou University, Lanzhou, 730000, China; Key Laboratory of Pan-third Pole Biogeochemical Cycling, Lanzhou University, Lanzhou, 730000, China; Chayu Monsoon Corridor Observation and Research Station for Multi-Sphere Changes, Lanzhou University, Chayu, 860600, China; State Key Laboratory of Advanced Environmental Technology, School of Environment, University of Science and Technology of China, Hefei, 230026, China; Center for Pan-third Pole Environment, Lanzhou University, Lanzhou, 730000, China; Key Laboratory of Pan-third Pole Biogeochemical Cycling, Lanzhou University, Lanzhou, 730000, China; Chayu Monsoon Corridor Observation and Research Station for Multi-Sphere Changes, Lanzhou University, Chayu, 860600, China; College of Ecology, Lanzhou University, Lanzhou, 730000, China; Center for Pan-third Pole Environment, Lanzhou University, Lanzhou, 730000, China; Key Laboratory of Pan-third Pole Biogeochemical Cycling, Lanzhou University, Lanzhou, 730000, China; Chayu Monsoon Corridor Observation and Research Station for Multi-Sphere Changes, Lanzhou University, Chayu, 860600, China; College of Ecology, Lanzhou University, Lanzhou, 730000, China; Center for Pan-third Pole Environment, Lanzhou University, Lanzhou, 730000, China; Key Laboratory of Pan-third Pole Biogeochemical Cycling, Lanzhou University, Lanzhou, 730000, China; Chayu Monsoon Corridor Observation and Research Station for Multi-Sphere Changes, Lanzhou University, Chayu, 860600, China; State Key Laboratory of Tibetan Plateau Earth System, Environment and Resources, Institute of Tibetan Plateau Research, Chinese Academy of Sciences, Beijing, 100101, China; University of Chinese Academy of Sciences, Chinese Academy of Sciences, Beijing, 100101, China; Center for Pan-third Pole Environment, Lanzhou University, Lanzhou, 730000, China; Key Laboratory of Pan-third Pole Biogeochemical Cycling, Lanzhou University, Lanzhou, 730000, China; Chayu Monsoon Corridor Observation and Research Station for Multi-Sphere Changes, Lanzhou University, Chayu, 860600, China

**Keywords:** archaea, provirus, antiviral system, anti-defense system, auxiliary metabolic gene

## Abstract

Archaea, the third domain of life, play critical roles in global biogeochemical cycles. However, archaeal proviruses integrated into host genomes remain largely unexplored. To bridge this gap, we conducted a large-scale mining of genomes spanning all presently known 21 archaeal phyla for their proviruses. We identified 770 archaeal proviruses across 12 archaeal phyla and 84 families, which clustered into 655 viral operational taxonomic units (vOTUs). Among these, 86.1% of the vOTUs were novel at the species level, and 69.3% could not be classified at the family level, substantially expanding the known diversity of archaeal viruses. Additionally, phylogenomic analysis supported the proposal of 16 putative novel viral families, further extending the current taxonomy landscape of archaeal viruses. Notably, 21.8% of the identified proviruses were predicted to adopt a lytic lifestyle, suggesting that these proviruses may retain the capacity to enter the lytic cycle under appropriate conditions. Host prediction indicated only 14 out of the 655 vOTUs might have potential across-lineage infection abilities. We detected 63 anti-defense genes encoded by 61 provirus genomes, such as anti-CRISPR and anti-RM, suggesting an ongoing evolutionary arms race between hosts and proviruses. However, only 10 auxiliary metabolic genes (AMGs) were identified, suggesting a limited impact of proviruses in the modulation of host metabolism through AMGs. This study establishes a systematic global genomic atlas of archaeal proviruses, advancing our understanding of their distribution and diversity while providing a foundation for future research into how proviruses regulate archaeal metabolism and ecosystem functioning.

## Introduction

Archaea, the third domain of life on Earth, represent a major group of microorganisms alongside Bacteria and Eukaryotes [1, 2]. The taxonomic classification of Archaea has been continuously revised and expanded over the past three decades. Advances in phylogenetic and genomic analyses have markedly increased the number of recognized archaeal phyla by 10-fold, expanding from the original two phyla (Euryarchaeota and Crenarchaeota) [1] to 21 phyla in the Genome Taxonomy Database (GTDB, version 226) [3], which are largely classified into three major superphyla: TACK (an acronym formed from the initials of the first four phyla discovered: Thaumarchaeota, Aigarchaeota, Crenarchaeota, and Korarchaeota), DPANN (an acronym formed from the initials of the first five phyla discovered: Diapherotrites, Parvarchaeota, Aenigmarchaeota, Nanohaloarchaeota and Nanoarchaeota), and Asgard [4–6]. In parallel, there have been significant advances in our understanding of archaeal metabolic capacities and functions. For example, non-Euryarchaeota capable of methanogenesis were isolated from oil fields and hot springs [7, 8]. A novel ammonia-oxidizing archaea genus, *Candidatus Nitrosomaritimum*, was identified in anaerobic intertidal aquifers of wetlands, which is capable of utilizing urea and reducing N_2_O, and is widely distributed in estuarine-coastal niches and marine sediments [9]. Novel lineages of TACK (Brockarchaeota) from hot spring and deep-sea hydrothermal vent sediments were found to be capable of mediating non-methanogenic anaerobic methylotrophy [10]. Meanwhile, functional annotation revealed the potential for aerobic hydrocarbon metabolism within a novel archaeal class of Syntropharchaeia in copper mine and groundwater environments [11]. Moreover, the acidophilic and thermophilic archaeon *Vulcanisaeta moutnovskia*, discovered in acidic hot springs, performs dissimilatory sulfate reduction [12], and Lokiarchaeon, a unique archaeal group that bridges the gap between prokaryotes and eukaryotes, exhibits the ability of homoacetogenesis in sulfidic marine sediments [13]. These findings highlight the key roles of Archaea in driving global biogeochemical cycling across diverse ecosystems and habitats [14] However, the diversity and ecological roles of archaeal proviruses remain largely unexplored.

Viruses are the most abundant biological entities on Earth and play essential roles in shaping microbial communities and driving biogeochemical cycles [15]. To date, the International Committee on Taxonomy of Viruses (ICTV, VMR_MSL41_v1) has officially established 82 archaeal viral families, with most (80.3%) of which are represented by isolates obtained from hyperthermophilic or hyperhalophilic hosts within the phyla Thermoproteota and Halobacteriota [16–18]. Archaeal proviruses are defined as viral genomes that have integrated into the genomic deoxyribonucleic acid (DNA) of the host cell [19]. The majority of known archaeal proviruses include head-tailed viruses and pleolipoviruses [19]. Recent metagenomic studies continue to expand the spectrum of archaeal viruses, adding 22 novel archaeal viral families from Halobacteriota, Thermoplasmatota, Thermoproteota, Asgardarchaeota, Nanoarchaeota, and DPANN phyla [19–22]. These discoveries have been enabled by diverse archaeal virus discovery strategies, including clustered regularly interspaced short palindromic repeats (CRISPR) spacer-guided identification [23], viral hallmark gene searches [24, 25], integrase-based mining [19, 26], and metagenomic recovery workflows [27, 28]. More recently, integrated viral identification pipelines such as geNomad [29] and VirSorter2 [30] have incorporated multiple complementary approaches, including viral hallmark gene detection, hidden Markov model (HMM)-based annotation, genomic feature analysis, and machine learning, enabling more systematic identification of viral sequences from genomes and metagenomes. Since these integrated approaches rely on different biological signals and detection strategies [29, 30], they are expected to recover partially distinct subsets of archaeal viruses and proviruses. Despite these advances, proviruses remain uncharacterized in the remaining eight archaeal phyla (e.g. Korarchaeota), making the diversity and biology of archaeal proviruses poorly understood.

During the long evolution of virus–host interactions, viruses have developed diverse strategies to ensure successful replication, including manipulating host metabolism and evading host immunity [31, 32]. One such strategy involves viral auxiliary metabolic genes (AMGs), which can enhance or redirect host metabolic processes to facilitate viral replication [31]. For instance, AMGs identified in marine Thaumarchaeota viruses encode a key subunit of the ammonia monooxygenase (*amoC*), a central enzyme in nitrification, potentially enhancing the host’s energy metabolism and viral replication during infection [33]. Meanwhile, persistent viral pressure has driven the evolution of sophisticated host defense systems, such as restriction–modification (RM) systems and CRISPR–Cas [34], which in turn has led to the emergence of diverse viral anti-defense mechanisms [35]. However, only a limited repertoire of anti-defense systems and AMGs has been identified in archaeal viruses to date [16, 36, 37], with a comprehensive assessment of anti-defense systems and AMGs encoded by archaeal proviruses remains lacking.

To fill these knowledge gaps in archaeal provirus diversity, classification, and virus–host interactions, we conducted a systematic genomic mining of currently available archaeal genomes. This study aimed to: (i) reveal the diversity, classification, and host range of archaeal proviruses; (ii) identify antiviral systems in archaeal hosts and associated anti-defense mechanisms encoded by proviruses; and (iii) characterize AMGs carried by archaeal proviruses and assess their potential metabolic roles. Our analysis reveals a vast and phylogenetically diverse array of archaeal proviruses, which encode a variety of anti-defense system and AMGs. These findings significantly expand the known diversity of archaeal proviruses and provide new insights into virus–host interactions in archaea.

## Materials and methods

### Archaea genome collection

This study collected a total of 25 530 archaeal genomes, comprising 24 840 genomes [including cultured isolates, metagenome-assembled genomes (MAGs), and single-amplified genomes (SAGs)] obtained from the NCBI GenBank database (November 2024), and 690 archaeal MAGs from the cryosphere environments of the Tibetan Plateau which have been published in our previous studies [38–40]. Following data collection, we performed quality screening and taxonomic validation on all archaeal genomes, details see Supporting Information Method S1. Ultimately, our study generated a non-redundant set of 21 989 medium- to high-quality archaeal genomes (completeness ≥50% and contamination ≤10%), with 21 593 from the NCBI GenBank database and 396 from our own. These archaea were classified into 21 phyla, 65 classes, 172 orders, 603 families, and 2075 genera (see Table S1 for details).

### Identification, quality checking, and clustering of proviruses

Provirus regions (≥5 kb) were first identified from archaeal genomes using geNomad v1.11.0 [29] and Virsorter2 v2.2.4 [30] with default parameters. Putative provirus sequences from the two pipelines were merged, and duplicate sequences were removed via an all-versus-all BLASTn analysis (parameters: -perc_identity 100 -qcov_hsp_perc 100 -evalue 1e-3). The resulting sequences were then processed with CheckV v1.0.1 (database v1.5) [41] for quality assessment, during which only proviruses containing viral hallmark genes were retained. The provirus genomes were further screened and retained based on the following criteria: (i) geNomad virus score ≥ 0.8 and either encode one virus hallmark (for example, terminase, capsid proteins, portal protein and so on) or with geNomad viral marker score ≥5.0, (ii) score >0.9 for VirSorter2, or score between 0.7–0.9 with at least one viral hallmark gene, (iii) high- or medium-quality CheckV completeness estimates or the presence of direct terminal repeats (DTRs). The archaeal sequence was considered to contain an integrated provirus only if viral regions were flanked by two host regions in this contig [20]. To ensure the origination of provirus from archaea, we manually checked the taxonomy of host regions by searching host proteins against the NCBI NR database using BLASTp [42]. Finally, the obtained proviruses were clustered at 95% average nucleotide identity (ANI) and 85% alignment fraction (AF) of the shortest proviruses using the anicalc.py and aniclust.py scripts provided along with the CheckV pipeline to generate non-redundant viral operational taxonomic units (vOTUs) [41].

### Lifestyle prediction of proviruses

The lifestyle of proviruses was predicted using four methods, details see Supporting Information Method S2. The combined result of more than two software was used as the final prediction result. Conflicting results between two software were resolved through sequential priority assignment to CheckV, gene annotation, VIBRANT [43], and PhaTYP [44] outputs.

### Taxonomic classification of viral operational taxonomic units

The obtained vOTUs were taxonomically classified using geNomad v1.11.0 [29], vConTACT3 v3.1.6 [45], VITAP v1.7.1 [46], PhaGCN3 v3.1, PhaGCN2 v2.3 [47], and viral genome homolog search method based on BLASTn comparison of the IMG/VR V4 database [48]. Details of the taxonomic procedure are provided in the Supporting Information Method S3.

To further clarify the family-level taxonomic affiliation of unclassified complete proviruses encoding major capsid proteins (MCPs) belonging to *Caudoviricetes*, a proteomic tree was constructed using ViPTree web server v4.0 [49] with default parameters. The input dataset comprised three components, as detailed in the Supporting Information Method S3. The orthologous fraction (OF) of proteins shared by viral genomes was estimated using CompareM (https://github.com/dparks1134/CompareM) (parameters: -evalue 1e-5 -identity 30%), and the input sequences were the same as those for ViPTree analysis. According to established classification criteria, viral lineages in the proteome-based phylogenetic tree with branch lengths ≥0.05 and sharing <10% OF with recognized families were designated as potential novel families [20, 50, 51].

### Comparison of provirus genomes and proteins to public databases

Archaeal provirus sequences and their encoded proteins were compared against the existing archaeal virus reference dataset, including IMG/VR v4 [48], NCBI Viral RefSeq (Release September 2025), and representative archaeal viruses reported in previous studies [19–21, 23–27, 37, 51–54]. First, all known archaeal virus sequences from these databases were clustered into vOTUs using the above method with 95% ANI and 85% AF. Next, the 655 vOTUs identified in our study were clustered against this reference set of vOTUs. Finally, the amino acid sequences of our archaeal vOTUs were predicted with Prodigal v2.6.3 [55] and subsequently clustered with the amino acid sequences of archaeal viruses from the reference archaeal viruses using CD-HIT v4.8.1 (parameters: -c 0.6 -G 0 -aS 0.8 -n 4) [56]. To further investigate the global distribution of archaeal proviruses, geographic and environmental information associated with viral sequences from the reference archaeal virus dataset that clustered with archaeal proviruses was manually retrieved.

### Identification of antiviral systems and provirus–host predictions

CRISPR-Cas genes and arrays were extracted from archaeal genomes using CRISPRcasFinder v4.3.2 [57], retaining those with evidence level 4 (repeat conservation index >70% and spacer identity <8%). To minimize potential false positives arising from mis-binning of CRISPR-containing contigs, CRISPR arrays were further curated. Specifically, only arrays located on contigs flanked on both sides by host regions were retained. The flanking regions were annotated using BLASTp against the NCBI NR database, and their taxonomic assignments were determined based on the taxonomy of the top BLASTp hits. In addition, the repeat sequences of the CRISPR array were queried against the NCBI NT database using BLASTn to verify their consistency with the assigned host genus. Only CRISPR arrays whose flanking regions and repeat sequences were both consistent with the assigned archaeal host lineage were retained. CRISPR–Cas systems in these contigs with CRISPR arrays were annotated by CRISPRCasTyper v1.8.0 [58]. Other antiviral defense systems in archaeal were detected using DefenseFinder v2.0.0 (parameters: --db-type gembase) [59, 60], updated with all known anti-phage systems in February 2025 (defense-finder-models v2.0.2).

To predict host–provirus interactions, we first performed BLASTn alignments between all proviruses and the archaeal CRISPR-spacer dataset. If a spacer generated an exact match (identity = 100%, coverage = 100%) with a provirus, we assigned the host associated with the spacer to the provirus. Furthermore, potential hosts for archaeal proviruses were also predicted using the integrated CRISPR method in iPHoP v1.4.1 (database: iPHoP_db_Jun25_rw) [61], with a confidence score cutoff of 95.

### Identification of anti-defense systems

The identification of anti-defense genes in proviruses was conducted using two approaches. (i) Anti-defense genes in the provirus were predicted using DefenseFinder (--antidefensefinder-only) [59, 60]. (ii) Anti-Prokaryotic Immune System (APIS) was selected as a reference database [62], which contains experimentally validated viral proteins that counteract prokaryotic immune systems and their associated protein families. DIAMOND BLASTp v0.9.14 (parameters: --evalue 1e-10 --id 30 --max-target-seqs 1) [63] and HMMER v3.4 (parameters: --domtblout --noali -E 1e-10) [64] were used to annotate the anti-defense genes from provirus sequences following the protocol provided by dbAPIS (https://bcb.unl.edu/dbAPIS/downloads/readme.txt).

### Protein function annotation and auxiliary metabolic genes identification

Protein-coding sequences of proviruses were predicted using Prodigal v2.6.3 (parameter: -p meta) [55], and the resulting protein sequences were annotated using InterProScan (v5.76-107.0) [65] and HHsearch v3.3.0 [66]. AMG prediction were performed using the DRAM-v pipeline [67] for proviruses encoding at least one viral hallmark gene, including MCP, terminase large subunit (TerL), or portal proteins. Details of the function annotation and AMGs identification procedure are provided in the Supporting Information Method S4. To enhance the confidence of AMG identification, we further performed manual curation to exclude genes potentially involved in essential viral processes, including viral invasion (i.e. glycoside hydrolases and peptidases involved in cell wall lysis), modification of viral components (i.e. glycosyl transferases, adenylyltransferases and methyltransferases that putatively involved in viral DNA, RNA, and structural proteins modification), viral replication, transcription, and translation (e.g. nucleotide metabolism, and ribosomal proteins), and folate biosynthesis [31, 68, 69].

### Phylogenetic analysis

The archaeal phylogenetic tree was constructed based on 53 archaeal concatenated marker genes generated by GTDB-Tk, using a maximum-likelihood approach implemented in IQ-TREE v3.0.1 (automatic model selection and 1000 bootstraps) [70].

To investigate the probable evolutionary origin of the AMGs, homolog sequences of AMGs were recruited from the NCBI nr database (Release October, 2025), and the top 20 hits with a bit score >50 were retained as reference sequences for the AMGs. The HK97 MCPs of 16 novel families and other reference archaeal viruses were merged into a single sequence set. AMGs and HK97 MCP sequence sets were separately aligned using MAFFT v7.525 (parameters: --maxiterate 1000 --localpair) [71], and trimmed using trimAl v1.5.0 [72] to remove poorly aligned regions. Maximum-likelihood trees were built using IQ-TREE with the above parameters and visualized using the iTOL online server [73]. We regard the sources of sequences that cluster closely with AMG in the phylogenetic tree as potential sources of the corresponding AMG.

Statistical analyses and visualization were performed as described in Supporting Information Method S5.

## Results

### The overall landscape of provirus elements in archaea

**Figure 1:**
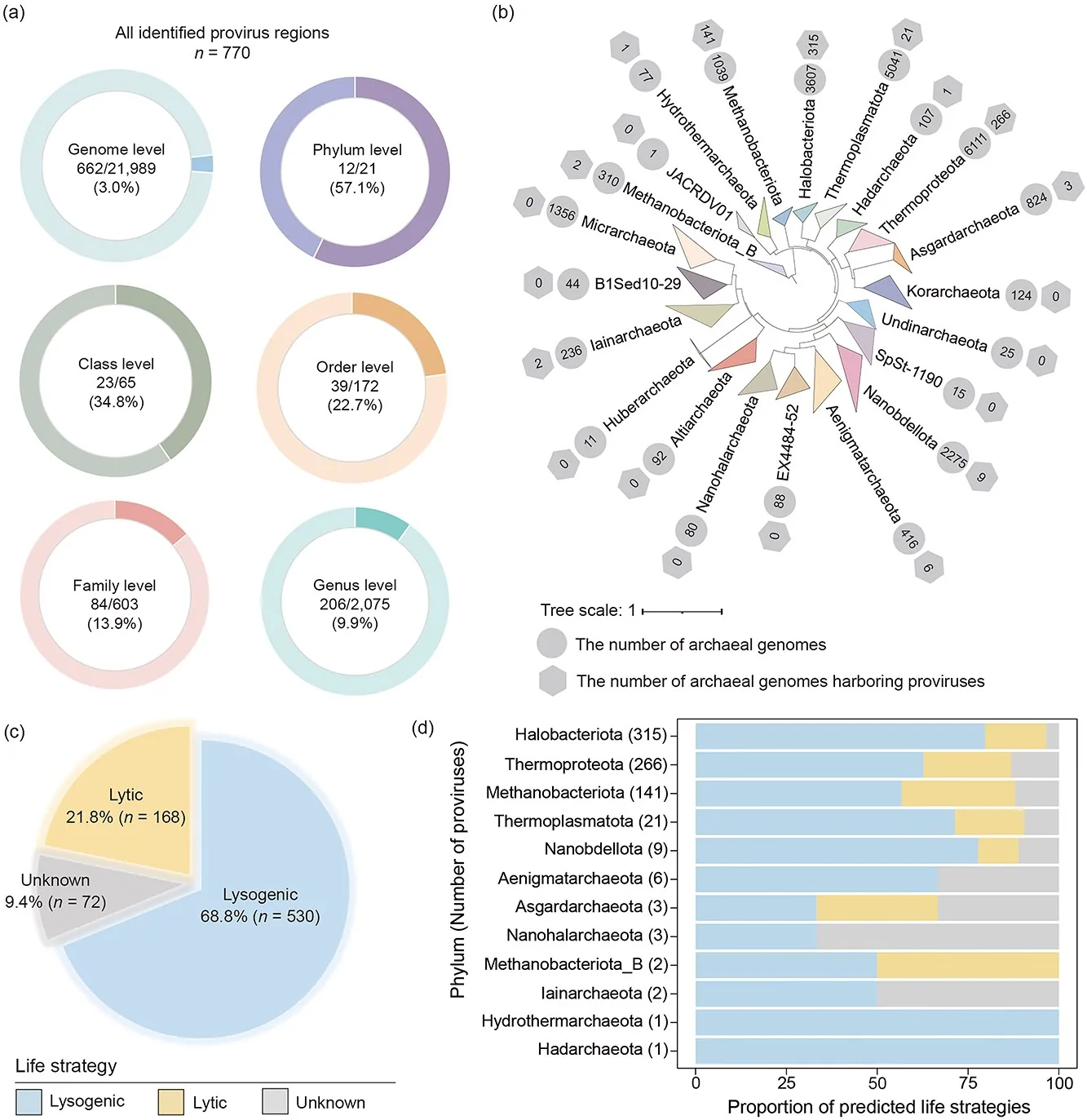
The overall landscape of archaeal proviruses. (a) The detection rates of all identified provirus regions at the genome level and the phylum, class, order, family, and genus levels. (b) Maximum-likelihood phylogenetic tree of 21 989 archaeal genomes, which was constructed based on 53 archaeal marker genes of GTDB-Tk. The gray circles indicate the number of archaeal genomes in the corresponding phylum, while the gray hexagons indicate the number of archaeal genomes carrying proviruses. (c) Proportion of lysogenic, lytic, and unknown in archaeal proviruses. (d) Proportion of predicted life strategies of archaeal proviruses across different phyla. The numbers in parentheses indicate the total number of proviruses in each phylum. Panels (c) and (d) share the same legend.

In total, 770 provirus regions (≥5 kb) were identified in 3.0% (662/21989) of the genomes (Table S2). These proviruses were distributed across 57.1% of the known archaeal phyla (12/21), 34.8% of classes (23/66), 22.7% of orders (39/172), 13.9% of families (84/603), and 9.9% of genera (206/2075) in the dataset (Fig. 1a; Table S3). At the phylum level, Halobacteriota contained the largest number of proviruses (315), followed by Thermoproteota (266) and Methanobacteriota (141) (Fig. 1b). Additionally, small number or no provirus were detected in several phyla with large numbers of genomes. For example, Thermoplasmatota (5041 genomes) and Nanobdellota (2275 genomes) collectively contained only 30 provirus regions, and no provirus was identified in Micrarchaeota (1356 genomes), Korarchaeota (124 genomes), and Altiarchaeota (92 genomes) (Fig. 1b). Parallel trends were observed at lower taxonomic ranks, including class, order, and family (Table S3). For instance, the families *Nitrosopumilaceae* (1078 genomes) and *Thalassarchaeaceae* (1005 genomes) harbor only five and no proviruses, respectively (Table S3).

The number of identified proviruses varied greatly among archaeal genomes, with ~86.6% (573) of genomes harboring only one provirus, while 89 genomes carried more than two proviruses (Fig. S1a). One extreme case was the MAG (GCA_963677865.2) of *Methanotrichaceae*, which contained seven provirus regions. Most of the predicted provirus regions (90.9%) are incomplete provirus fragments, while 70 provirus regions are marked as intact proviruses (evaluated as “complete 100%” by CheckV), distributed in 66 (0.3%) genomes and 15 (2.5%) archaeal families (Table S4; Fig. S1b). Among these 66 genomes, the 62 (93.9%) harbored one intact provirus, and four genomes contained two intact proviruses each (Table S4).

Besides distribution patterns, archaeal proviruses also exhibited pronounced variation in genomic features, with sizes ranging from 5.4 kb to 156.5 kb and an average length of 29.8 ± 23.2 kb (median ± interquartile range). Comparative analyses of the three host phyla containing >100 proviruses showed that both provirus and host genome sizes were significantly smaller in Thermoproteota than those in Halobacteriota and Methanobacteriota (*P* < 0.001) (Fig. S1c). In addition, archaeal provirus genomes also exhibited a wide range of Guanine-Cytosine (GC) (37.4%–70.7%). When analyzed by host phylum, provirus GC content generally matched the GC content of their archaeal hosts, with Halobacteriota representing a notable exception in which provirus GC content was significantly lower than that of the host genomes (*P* < 0.001) (Fig. S1d).

Interestingly, life strategy prediction indicated a substantial proportion of (168; 21.8%) proviruses with lytic lifestyles relative to the predominance of lysogenic lifestyles (530; 68.8%) (Fig. 1c). Among the three phyla with the largest numbers of proviruses, lysogenic lifestyles were dominant (56.7%–79.7%), whereas lytic proviruses accounted for 16.8%–31.2%, reflecting widespread lytic potential (Fig. 1d). Thermoplasmatota and Nanobdellota exhibited similar patterns, with lysogenic proviruses remaining dominant (71.4%–77.8%). In contrast, phyla represented by a small number of proviruses showed large variations, including exclusive lysogenic proviruses in Hadarchaeota and Hydrothermarchaeota and equal proportions of lysogenic and lytic proviruses in Methanobacteriota_B (Fig. 1d).

### Diversity, taxonomy, and genomic features of archaeal proviruses

To explore the diversity of archaeal proviruses, we performed a clustering analysis on the 770 identified proviruses and obtained 655 vOTUs. Overall, 489 vOTUs (74.7%) were classified to known taxa (Fig. S2;  Table  S5). These proviruses spanned four DNA virus realms: *Duplodnaviria* (68.5%, *n* = 449), *Singelaviria* (1.5%, 10), *Varidnaviria* (0.5%, 3), and *Pleomoviria* (0.2%, 1) (Fig. S2;  Table  S5). At the class level, the majority (68.5%) of vOTUs were assigned to *Caudoviricetes* of the phylum *Uroviricota* (Fig. S2). Only 201 (30.7%) vOTUs could be resolved to the family level, indicating high genetic diversity among archaeal proviruses and the presence of numerous previously undetected novel viruses. These vOTUs were distributed in 21 different families, mainly including *Graaviviridae* (49, 7.5%), *Kairosviridae* (38, 5.8%), *Fuselloviridae* (22, 3.6%), and *Hafunaviridae* (20, 3.1%) (Fig. S2). Among the 66 vOTUs represented by complete viral genomes, 22 remained unclassified at the family level but could be assigned to *Caudoviricetes*. Most of these 22 vOTUs were represented by a single provirus genome, whereas only two vOTUs (GCA_000698785.1_CP007536.1 and GCA_023699475.1_default_2) contained two complete provirus genomes each (Table S4). MCPs were detected in all but one of these 22 vOTUs (GCA_963677865.2_contig_2_4) (Table S6).

**Figure 2:**
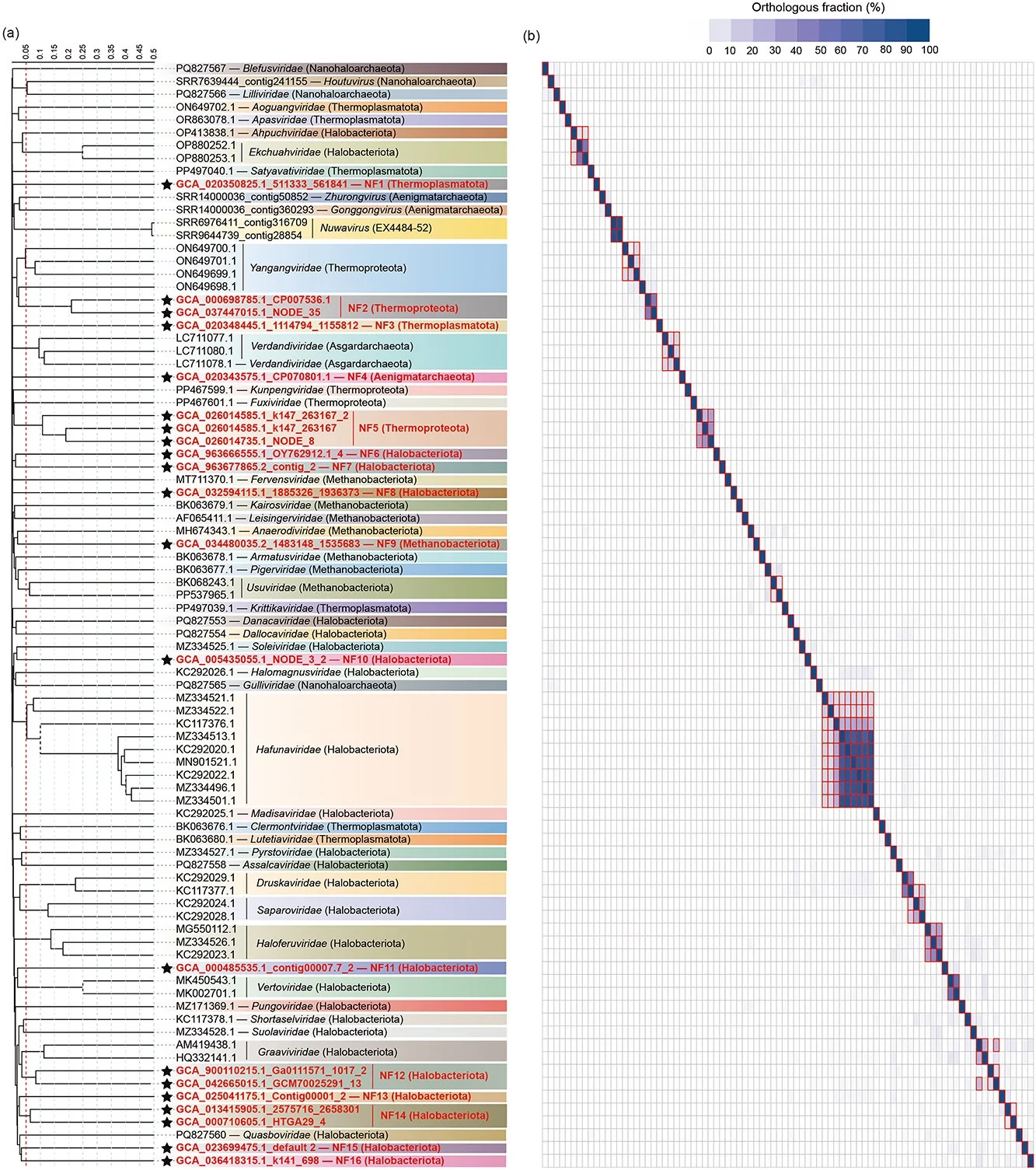
Genome-based taxonomic assignment of archaeal proviruses. (a) The proteomic phylogenetic tree. Complete archaeal provirus genomes are marked with black pentagrams and red font. Reference viruses are shown in black font, with their hosts in brackets. Red dashed lines in the proteomics phylogenetic tree denote the branch length for family-level demarcation (0.05). (b) Heatmap of OFs of the proviruses. Orthologous protein fractions ≥10% are denoted by the boxes with red borders.

To further elucidate the taxonomic classification of these unclassified proviruses, 21 MCP-encoding complete vOTUs were selected for phylogenetic analysis. Both whole genome proteome and MCPs based phylogenetic analysis consistently showed that these complete vOTUs formed 16 distinct branches that were phylogenetically distinct from the reference archaeal viruses (Fig. 2; Fig. S3). Protein-sharing network analysis using vConTACT3 also showed that these vOTUs formed discrete viral clusters separated from known viral taxa, with the exception of four vOTUs (Table S7). Specifically, GCA_023699475.1_default_2 (NF15) and GCA_042665015.1_GCM70025291_13 (NF12) exhibited network-level proximity to members of the family *Graaviviridae*, whereas GCA_000485535.1_contig00007.7_2 (NF11) and GCA_013415905.1_2575716_2658301 (NF14) showed associations with *Vertoviridae* and *Pyrstoviridae*, respectively (Table S7). Nevertheless, all these four vOTUs shared <10% OF of proteins with their closest relatives and did not cluster into the same branch in the phylogenetic tree (Fig. 2; Fig. S3). Given that all 16 identified viral groups consistently satisfy established taxonomic criteria for family-level differentiation, including proteome-based branch lengths of ~0.05 and OF values below 10% [20, 50, 51], these 16 groups likely represent putative novel viral families, provisionally designated as NF1 to NF16 (Fig. 2; Table S8).

All 16 viral groups encoded hallmark proteins of head–tail viruses, including HK97-like MCP, portal proteins, terminase large and small subunits, and multiple tail-associated components involved in virion assembly and host recognition (Fig. S4;  Table  S6). Beside structural and morphogenesis proteins, at least one gene associated with viral genome replication and packaging was identified in 13 archaeal families, with NF10 encoding DNA polymerase, DNA helicase, exonuclease, and His–Asn–His (HNH) endonuclease (Fig. S4;  Table  S6). DNA-binding proteins encoded by these viral groups showed diverse structural domains, including ribbon–helix–helix (RHH), helix–turn–helix (HTH), winged-helix and zinc finger motifs (Table S6), likely involved in regulating viral replication and host gene expression [74]. Proteins related to transcription were also detected, including ribonuclease P (RNase P) in two proviruses and RNA polymerase in three proviruses. In addition, five proviruses encode translation-associated components, such as ribosomal proteins S6e, L15, L15e, and L30, translation initiation factor IF1, tRNA-binding proteins, and multiple tRNA synthetases or ligases (Table S6), indicating that these archaeal proviruses harbor diverse machineries of host-interacting functions beyond virion assembly.

### Novelty, host range, and geographic distribution of archaeal proviruses

**Figure 3:**
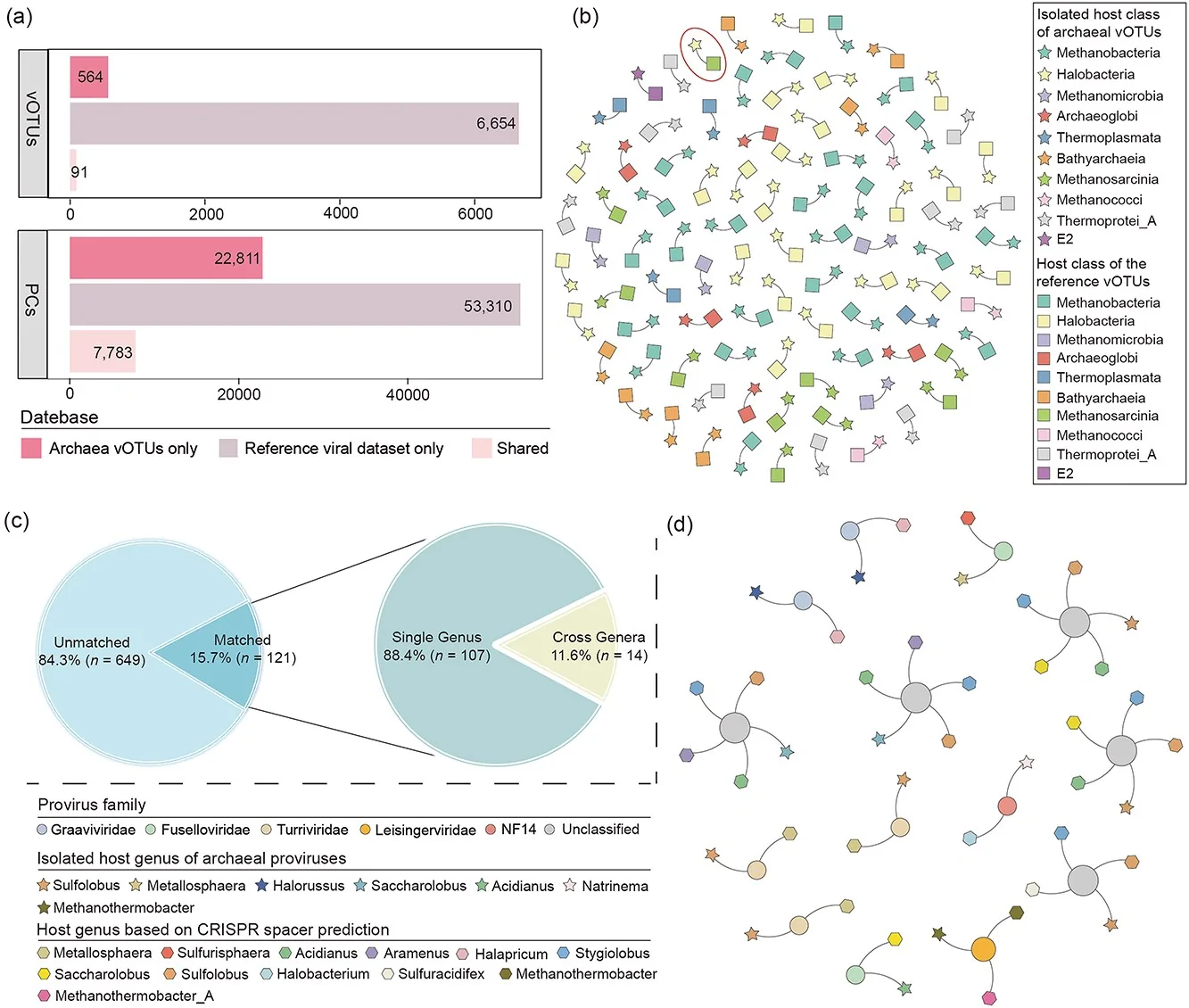
Novelty, host range, and host–virus interactions of the archaeal proviruses. (a) Number of vOTUs and their PCs in the archaeal provirus dataset, the reference viral dataset, and shared by the databases. The reference viral dataset includes previously published archaeal virus genomes, IMG/VR, and NCBI Viral RefSeq. (b) Integrative association diagram between the isolation host class of vOTUs identified in this study and the host class of the reference vOTUs. Stars represent the host of vOTUs identified in this study; squares represent the host of the reference vOTUs; and colors indicate archaeal class. Ellipses highlight the hosts associated with vOTU that potentially exhibit cross-class infection. (c) The CRISPR spacer sequence targeting situation of archaeal proviruses. The left chart shows that the vast majority (84.3%) of proviruses were not targeted by any spacers. The right chart details the host range of the targeted provirus. (d) This network depicts the relationship between proviruses and their hosts, including proviruses from isolated archaeal genomes, as well as host based on CRISPR spacer predictions. Circles represent proviruses, stars represent host of vOTUs identified in this study, and hexagons represent hosts of CRISPR spacer predictions. Colors indicate provirus family or archaeal genera.

Our provirus dataset significantly expands the known diversity of archaeal viruses. At the vOTU level, the majority of vOTUs (564, 86.1%) were unique to our dataset, with only 91 vOTUs shared with public databases (Fig. 3a). At the protein cluster (PC) level, we identified 22 811 novel PCs, whereas only 7783 PCs were shared with public databases (Fig. 3a).

Among these shared vOTUs, host classifications of proviruses were generally consistent within each vOTU cluster. The only exception was GCA_017874375.1_Ga0451180_02_2, which contained provirus sequences associated with Methanosarcina and Halobacteria genomes, suggesting potential infection capacity of it for both lineages (Fig. 3b). In addition, host prediction analysis revealed that 121 proviruses were targeted by CRISPR spacers. Among them, 88.4% (*n* = 107) were linked to spacers from a single genus, while the remaining 11.6% (*n* = 14) were linked to more than one distinct genus (Fig. 3c; Table S9). Among these, the unclassified proviruses (GCA_000968355.2_CP011055.2, GCA_003852095.1_CP033240.1, GCA_001560375.1_scaffold2 and GCA_001560385.1_scaffold2) were each found to link with 4 genera. Interestingly, among proviruses with spacer linkages, 10 were targeted by CRISPR spacers from a genus other than their own (Fig. 3d; Table S9).

**Figure 4:**
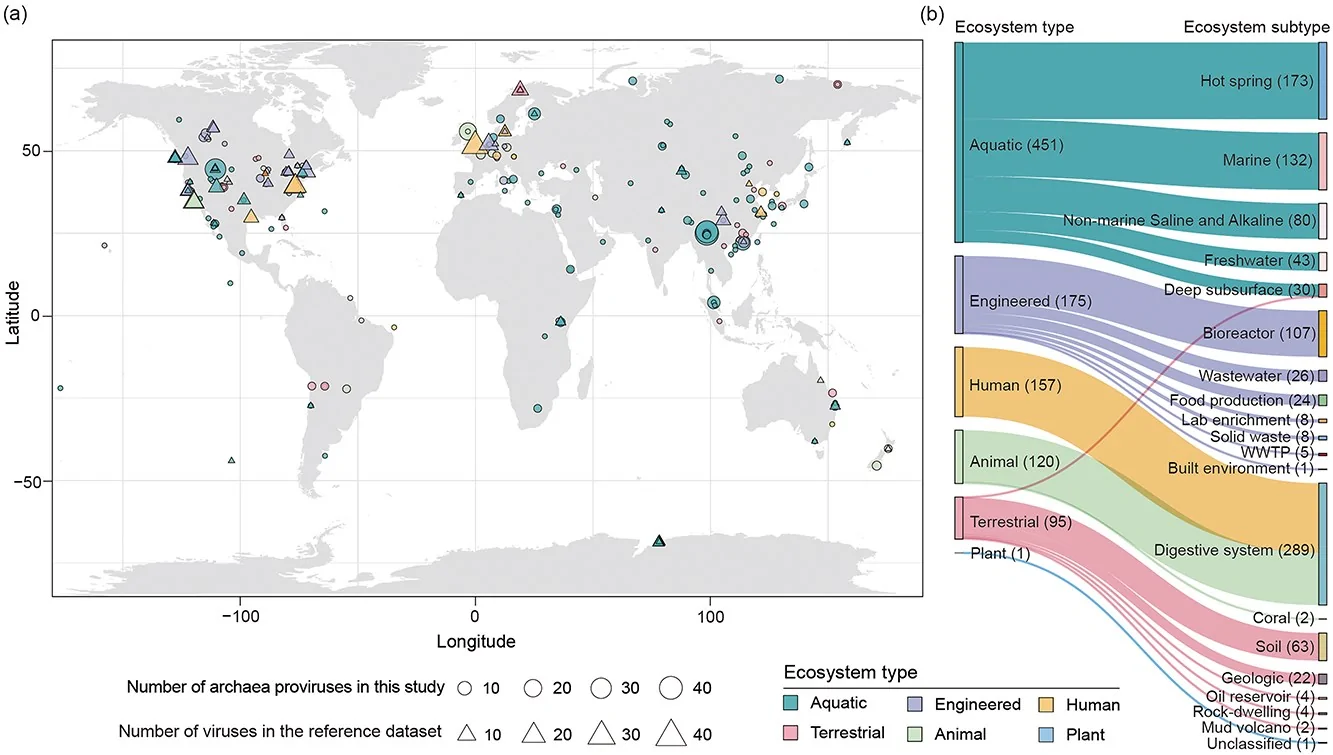
The global geographic distribution and environmental origins of archaeal proviruses and related viruses. (a) The geographic distribution of archaea carrying proviruses and viruses clustered with archaeal proviruses in IMG/VR, NCBI RefSeq, and previous studies. Only samples with available geographic coordinates are shown. Circles represent the number of archaeal proviruses, whereas triangles indicate the number of related viral sequences from the reference dataset. Symbol size is proportional to the number of samples, and colors denote environmental categories. (b) The number of archaeal proviruses and related viral sequences across environmental types and subtypes (shown in parentheses).

Comparative analysis of viral sequences from public databases revealed the widespread distribution of the archaeal proviruses detected here across diverse global ecosystems, including aquatic (*n* = 451), engineered (175), human-associated (157), animal-associated (120), terrestrial (95), and plant (1) environments (Fig. 4). The majority of sequences were detected in digestive systems (275), hot springs (173), marine environments (129), and bioreactors (104), with additional occurrences in soil (62), freshwater (41), and deep subsurface environments (29) (Fig. 4).

### Anti-defense systems of archaeal proviruses

**Figure 5:**
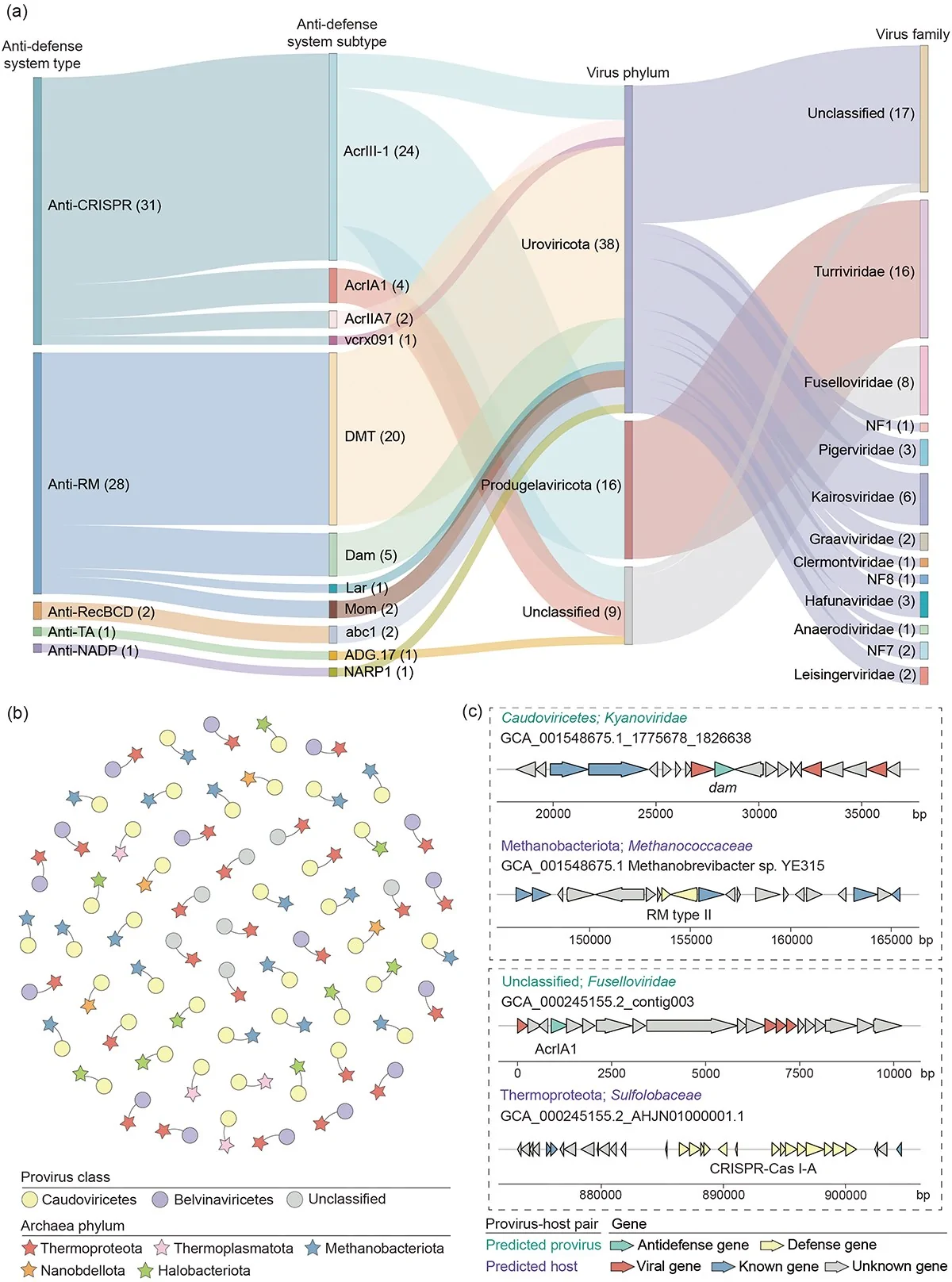
Interactions between archaeal provirus anti-defense systems and host antiviral systems. (a) Anti-defense genes identified in archaeal proviruses, showing the types of anti-defense systems and corresponding viral taxonomy. (b) A network diagram of 58 provirus–host pairs, where the provirus contains anti-defense genes and the corresponding host possesses antiviral system. Circles represent proviruses, stars represent archaeal hosts, and colors indicate provirus class or archaeal phylum. (c) Genomic architecture examples of provirus gene clusters containing anti-defense systems and their corresponding host containing antiviral systems.

Viruses can circumvent or block host defense pathways and ensure successful infection by encoding an arsenal of anti-defense proteins, which have been reported to span at least 25 types [62, 75]. Here, we detected genes related to 63 anti-defense systems in 8.2% of provirus genomes (*n* = 61), which were categorized into five types and 11 subtypes (Fig. 5a). Among these, anti-CRISPR (Acr) types were the most prevalent both in number and diversity (31 genes, 4 subtypes), followed by anti-RM (28 genes, 4 subtypes), anti-RecBCD (two genes, one subtype), anti-TA (one gene) and anti-NADP (one gene) (Fig. 5a). Furthermore, most of proviruses (96.7%, *n* = 59) encode a single anti-defense system, while only two proviruses contain two types of anti-defense systems.

We identified 58 provirus–host pairs in which the host encoded an antiviral system and the associated provirus carried the anti-defense system (Fig. 5b; Table S10; Supplemental Information Result 1). These interactions were predominantly observed between proviruses of the class *Caudoviricetes* and their hosts, including members of the phyla Methanobacteriota (18 pairs) and Halobacteriota (9 pairs) (Fig. 5b). In contrast, all hosts linked to *Belvinaviricetes* proviruses carrying anti-defense systems belonged to the phylum Thermoproteota. Among these pairs, only seven proviruses possess anti-defense systems targeting their respective host’s defense machineries. Specifically, three *Caudoviricetes* proviruses encoded DNA adenine methylase *dam* gene predicted to counteract the RM antiviral system of their host belonging to the family *Methanobacteriaceae* [76]. Additionally, four *Fuselloviridae* proviruses encoded AcrIA1 proteins, which is predicted to inhibit type I-A CRISPR-Cas systems of their *Sulfolobaceae* hosts (Fig. 5c; Table S10).

### Auxiliary metabolic genes of archaeal proviruses

We identified 10 AMGs in 10 proviruses, which potentially participate in seven biological pathways associated with metabolism (six pathways) and environment information processing (one pathway) (Table S11). These pathways spanned five functional categories, including metabolism of amino acids (5, 50.0%), cofactors and vitamins (2, 20%), carbohydrates (1, 10.0%), energy (1, 10.0%), and membrane transport (1, 10.0%) (Table S11). Among these, amino acid metabolism-related AMGs include *dapA*, encoding 4-hydroxy-tetrahydrodipicolinate synthase involved in lysine biosynthesis, and *metY*, encoding O-acetylhomoserine (thiol)-lyase associated with cysteine and methionine metabolism. In addition, AMGs involved in cofactor and vitamin metabolism included *iscS* (cysteine desulfurase) and *pdxI* (pyridoxine 4-dehydrogenase), which are associated with thiamine and vitamin B6 metabolism, respectively. A carbohydrate metabolism-related AMG, *gmd* (GDP-mannose 4,6-dehydratase), involved in GDP-mannose conversion, and an energy metabolism-associated gene, *narC*, encoding a cytochrome component of the nitrate reductase complex, were also identified (Table S11). We observed functional redundancy between AMGs and host metabolic genes, as revealed by comparing predicted AMGs with their host genomes. Specifically, six AMGs shared homology with host genes (>30% amino acid identity over 50% of viral query sequence length) (Table S11). Conversely, three AMGs showed no detectable homology to host genes, yet phylogenetic analysis revealed that some of them were consistent with the evolutionary origins of their hosts. For instance, AMGs encoding NarC exhibit a phylogenetic affiliation with the Halobacteriota, the same phylum as their hosts (Table S11;  Fig.  S5).

## Discussion

Compared to bacteria, research on archaeal proviruses is very limited. Through in-depth mining of public data, we have constructed the most comprehensive archaeal provirus genome dataset to date. The overall provirus recovery rate (~3%) was higher than that reported in previous large-scale archaeal genome mining studies [19, 20], likely reflecting the improved sensitivity of our provirus identification pipeline. In the latest ICTV classification (VMR_MSL41_v1), archaeal viruses include 5 realms, 6 classes, and 82 families [17]. The archaeal viruses that have been identified belong primarily to the families *Sphaerolipoviridae, Rudiviridae*, and *Demerecviridae* [48]. Recent archaeal virus research has discovered 22 new viral families across multiple archaeal phyla [19–22]. For example, the first successful cultivation of a virus belonging to a new family “Fylgjaviridae” infecting a novel Asgard strain of *Ca.* Lokiarchaeum ossiferum B36 were reported recently [22]. In addition, 14 potential novel viral families were identified across four archaeal phyla, namely Halobacteriota, Thermoplasmatota, Thermoproteota, and Nanoarchaeota [19], while seven viral families, including *Zhurongvirus, Gonggongvirus, Houtuvirus, Houyivirus, Nuwavirus, Kirinvirus*, and *Ditingvirus*, were identified within the DPANN superphylum [20]. The 655 archaeal provirus vOTUs discovered in this study cover 4 realms, 4 classes, and 38 families (Table S5). Of these archaeal proviruses, over 86.1% are new viral species, and 21 vOTUs belong to 16 new viral families (Fig. 2; Table S7). From a host perspective, the 655 vOTUs belong to 84 archaeal families, 30 (containing 43 vOTUs) of which have no viruses been previously reported. Among the 16 new viral families, the new family NF3 infects the archaeal family GCA-020350865, belonging to the class Thermoplasmata, an archaeal group with no viruses been previously reported. Therefore, our work reveals the hidden diversity of archaeal proviruses and expands our understanding of existing viruses. However, viral sequences within the 16 proposed families or belonging to host groups with virus first reported here contain only 1 or 2 members, indicating that the genetic diversity of archaeal proviruses remains largely under sampled. Furthermore, comparative database analysis shows that archaeal proviruses are widely distributed across different habitats globally, and their hosts involve groups participating in processes such as biogeochemical cycling, indicating a broad and significant potential impact of archaeal proviruses on biogeochemical cycles, but this impact requires further investigation.

Notably, 21.8% of the identified proviruses were predicted to adopt a lytic lifestyle, suggesting that these proviruses may retain the potential to enter the lytic cycle under specific environmental or cellular stress conditions, ultimately leading to host cell lysis and the release of viral particles. For example, the integrated haloarchaeal provirus SNJ1 can be induced by mitomycin C treatment, resulting in a marked increase in viral production [77], whereas the integrated proviruses APSV1 and APOV1 in *Aeropyrum pernix* have been shown to initiate viral replication following changes in host growth conditions [78]. In addition, DNA damage has been shown to trigger the induction of the temperate archaeal virus SNJ2 through a virus-encoded DNA damage signaling pathway [79]. These predicted lytic proviruses were detected across diverse archaeal host groups and environmental habitats (Fig. 1d; Table S4), suggesting that the potential for lytic induction may be widespread among archaeal proviruses.

Viral influence on host metabolism through AMGs is one pathway of virus–host interactions. Previous studies have found that archaeal viruses encode various types of AMGs [19, 33, 80, 81]. These AMGs are involved in multiple metabolic pathways, including the carbon, nitrogen, and sulfur cycling. For example, AMGs such as sulfite oxidase (SUOX) and glutamate–cysteine ligase (GshA) have been identified in Halobacteriota, suggesting potential roles in sulfur metabolism, and redox homeostasis [19]. In Thermoproteota, proviruses have been found to encode AMGs including phycocyanobilin lyase subunit alpha (CpcE), tyrosine decarboxylase (MfnA), and UDP-glucose 4-epimerase (GALE), which are involved in energy metabolism, amino acid metabolism, and carbohydrate metabolism, respectively [19, 80]. In addition, viruses associated with DPANN archaeal encode AMGs related to carbon utilization (e.g. pyruvate metabolism), sulfur reduction, and organic nitrogen processing, including AMGs coding enzymes such as pyruvate dehydrogenase and threonine/serine dehydratases, which may redirect host carbon flux toward viral replication [81]. Some AMGs are also linked to cell surface modification, potentially influencing virus–host attachment and interactions [81]. These findings collectively indicate that archaeal viruses may influence a wide range of host metabolic processes, potentially enhancing host adaptability to diverse environmental conditions. However, contrary to our expectations and existing research results, we found that archaeal proviruses, similar to RNA viruses, rarely carrying AMGs [82]. This may be partially due to the high proportion of incomplete provirus genomes, which may have contributed to reduced AMG recovery. In addition, our study focused only on proviruses, whereas previous studies also included free viral genomes recovered from metagenomic datasets, which may also contribute to differences in the identification AMGs. Nevertheless, our results suggest that the ability of archaeal proviruses to mediate horizontal transmission of host genes and influence host metabolism through AMGs is relatively limited.

Although our dataset uncovered numerous novel and unclassified archaeal proviruses, we expect that many more remain undiscovered, and their accurate identification and taxonomic classification remain challenging. This is primarily due to the underrepresentation of archaeal genomes in public databases, the high divergence of archaeal viral sequences, and the scarcity of well-characterized reference genomes [83]. Specifically, the provirus identification strategy adopted in this study, which relied on geNomad and VirSorter2 followed by stringent filtering for integrated proviruses, may preferentially recover proviruses sharing detectable similarity with currently available reference viruses. Consequently, highly divergent archaeal viruses, including members of poorly represented viral groups such as *Adnaviria* or viruses associated with archaeal lineages lacking sufficient reference genomes, may have been underestimated or missed. This may also explain why no proviruses were recovered from several archaeal phyla for which viruses have previously been reported from metagenomic studies [20]. Notably, some previously reported proviruses were not recovered, or only partially recovered, from the corresponding archaeal genomes in our analysis (Table S1), which may partly reflect differences in viral identification strategies and filtering criteria among studies. In addition, the completeness of provirus sequences may influence the interpretation of genome size distributions, as well as the accuracy of lifestyle prediction and taxonomic classification, because fragmented proviruses may not represent the full genome and can lack hallmark genes required for reliable inference.

Similarly, although our pipeline successfully recovered previously reported archaeal anti-defense systems, such as Acr and anti-TA [36], the number of predicted anti-defense genes remained relatively small. This may be because current anti-defense databases are largely derived from bacterial phages, whereas experimentally validated archaeal anti-defense proteins remain extremely limited. As a result, highly divergent or archaeal-specific anti-defense proteins may be difficult to identify using existing databases, leading to an underestimation of their true diversity in archaeal proviruses. Moreover, the co-occurrence of defense and anti-defense genes in host and provirus genomes should be interpreted with caution, as it does not necessarily indicate direct functional interactions [35]. Addressing these difficulties is crucial, as studying archaeal viruses is not only essential for understanding their roles in host population regulation, horizontal genetic exchange, and biogeochemical cycling, but also holds promise for uncovering novel viral mechanisms and evolutionary trajectories.

To advance this field, future efforts should prioritize long-read sequencing, single-cell genomics, and targeted viral enrichment to improve provirus discovery, alongside machine-learning approaches for enhanced viral detection and functional annotation [83, 84]. Integrating large-scale ecological surveys with experimental validation of auxiliary metabolic and anti-defense genes will be key to elucidating virus–host interactions. Tackling these challenges and leveraging emerging technologies will be essential for a comprehensive understanding of the diversity, ecological roles, and evolutionary significance of archaeal viruses.

## Conclusion

In summary, this study provides a comprehensive analysis of archaeal proviruses and maps their complete genomic landscape, elucidating their taxonomic diversity, host interactions, and functional potential. We found that archaeal proviruses are not only widely distributed and highly diverse but also harbor a large number of unexplored lineages. Host range analysis revealed that some proviruses exhibit broad infectivity across archaeal lineages. The discovery of antiviral and anti-defenses systems underscores the ongoing evolutionary arms race between archaeal hosts and their proviruses. In addition, AMGs were detected and found to be involved in metabolic processes such as carbon and energy metabolism, suggesting that proviruses may indirectly modulate biogeochemical cycling through metabolic reprogramming of their hosts. These findings significantly advance our understanding of archaeal provirus diversity and provide a foundational resource for further exploring virus–host dynamics and the ecological impacts of Archaea.

## Supplementary Material

Supplementary_material_ycag243

## Data Availability

The present study did not generate codes, and mentioned tools used for the data analysis were applied with default parameters unless specified otherwise. The archaeal provirus genomes generated in this study have been deposited in Figshare (https://doi.org/10.6084/m9.figshare.32899136).

## References

[1] Woese CR, Kandler O, Wheelis ML. Towards a natural system of organisms: proposal for the domains archaea, bacteria, and eucarya. *Proc Natl Acad Sci USA* 1990;87:4576–9. 10.1073/pnas.87.12.4576 2112744 PMC54159

[2] Spang A, Saw JH, Jorgensen SL et al. Complex archaea that bridge the gap between prokaryotes and eukaryotes. *Nature* 2015;521:173–9. 10.1038/nature14447 25945739 PMC4444528

[3] Parks DH, Chuvochina M, Rinke C et al. GTDB: an ongoing census of bacterial and archaeal diversity through a phylogenetically consistent, rank normalized and complete genome-based taxonomy. *Nucleic Acids Res* 2022;50:D785–94. 10.1093/nar/gkab776 34520557 PMC8728215

[4] Castelle CJ, Banfield JF. Major new microbial groups expand dversity and alter our understanding of the tree of life. *Cell* 2018;172:1181–97. 10.1016/j.cell.2018.02.016 29522741

[5] Spang A, Caceres EF, Ettema TJG. Genomic exploration of the diversity, ecology, and evolution of the archaeal domain of life. *Science* 2017;357:eaaf3883. 10.1126/science.aaf3883 28798101

[6] Hug LA, Baker BJ, Anantharaman K et al. A new view of the tree of life. *Nat Microbiol* 2016;1:16048. 10.1038/nmicrobiol.2016.48 27572647

[7] Wu KJ, Zhou L, Tahon G et al. Isolation of a methyl-reducing methanogen outside the Euryarchaeota. *Nature* 2024;632:1124–30. 10.1038/s41586-024-07728-y 39048829

[8] McKay LJ, Dlakic M, Fields MW et al. Co-occurring genomic capacity for anaerobic methane and dissimilatory sulfur metabolisms discovered in the Korarchaeota. *Nat Microbiol* 2019;4:614–22. 10.1038/s41564-019-0362-4 30833730

[9] Zhao Z, Qin W, Li L et al. Discovery of *Candidatus Nitrosomaritimum* as a new genus of ammonia-oxidizing archaea widespread in anoxic saltmarsh intertidal aquifers. *Environ Sci Technol* 2024;58:16040–54. 10.1021/acs.est.4c02321 39115222

[10] De Anda V, Chen LX, Dombrowski N et al. Brockarchaeota, a novel archaeal phylum with unique and versatile carbon cycling pathways. *Nat Commun* 2021;12:2404. 10.1038/s41467-021-22736-6 33893309 PMC8065059

[11] Leu AO, Woodcroft BJ, McIlroy SJ et al. Potential for aerobic hydrocarbon oxidation in archaea. *Nat Commun* 2025;16:9188. 10.1038/s41467-025-64223-2 41102171 PMC12533140

[12] Chernyh NA, Neukirchen S, Frolov EN et al. Dissimilatory sulfate reduction in the archaeon ‘*Candidatus* Vulcanisaeta moutnovskia’ sheds light on the evolution of sulfur metabolism. *Nat Microbiol* 2020;5:1428–38. 10.1038/s41564-020-0776-z 32807893

[13] Orsi WD, Vuillemin A, Rodriguez P et al. Metabolic activity analyses demonstrate that Lokiarchaeon exhibits homoacetogenesis in sulfidic marine sediments. *Nat Microbiol* 2020;5:248–55. 10.1038/s41564-019-0630-3 31873205

[14] Baker BJ, De Anda V, Seitz KW et al. Diversity, ecology and evolution of archaea. *Nat Microbiol* 2020;5:887–900. 10.1038/s41564-020-0715-z 32367054

[15] Breitbart M . Marine viruses: truth or dare. *Annu Rev Mar Sci* 2012;4:425–48. 10.1146/annurev-marine-120709-142805 22457982

[16] Krupovic M, Cvirkaite-Krupovic V, Iranzo J et al. Viruses of archaea: structural, functional, environmental and evolutionary genomics. *Virus Res* 2018;244:181–93. 10.1016/j.virusres.2017.11.025 29175107 PMC5801132

[17] Lefkowitz EJ, Dempsey DM, Hendrickson RC et al. Virus taxonomy: the database of the international committee on taxonomy of viruses (ICTV). *Nucleic Acids Res* 2018;46:D708–17. 10.1093/nar/gkx932 29040670 PMC5753373

[18] Krupovic M, Baquero DP, Bignon EA et al. Summary of taxonomy changes ratified by the international committee on taxonomy of viruses (ICTV) from the archaeal viruses subcommittee, 2025. *J Gen Virol* 2025;106:002117. 10.1099/jgv.0.002117 40711890 PMC12451623

[19] Xu TQ, Ni YM, Li HL et al. Discovery and characterization of complete genomes of 38 head-tailed proviruses in four predominant phyla of archaea. *Microbiol Spectrum* 2025;13:e00492–24. 10.1128/spectrum.00492-24 PMC11705971 39545734

[20] Wu ZZ, Liu SF, Ni JR. Metagenomic characterization of viruses and mobile genetic elements associated with the DPANN archaeal superphylum. *Nat Microbiol* 2024;9:3362–75. 10.1038/s41564-024-01839-y 39448846

[21] Zhou YF, Gutiérrez-Preciado A, Liu Y et al. Viruses and virus satellites of haloarchaea and their nanosized DPANN symbionts reveal intricate nested interactions. *Nat Microbiol* 2025;10:2673–85. 10.1038/s41564-025-02149-7 41107452

[22] Maslać N, Ponce-Toledo RI, Rodrigues-Oliveira T et al. Active virus–host system in a Lokiarchaeon culture. bioRxiv. 2026;705702. 10.64898/2026.02.13.705702

[23] Duan CH, Liu Y, Liu Y et al. Diversity of *Bathyarchaeia* viruses in metagenomes and virus-encoded CRISPR system components. *ISME Commun* 2024;4:ycad011. 10.1093/ismeco/ycad011 38328448 PMC10848311

[24] Laso-Pérez R, Wu FB, Crémière A et al. Evolutionary diversification of methanotrophic ANME-1 archaea and their expansive virome. *Nat Microbiol* 2023;8:231–45. 10.1038/s41564-022-01297-4 36658397 PMC9894754

[25] Valentin-Alvarado LE, Shi LD, Appler KE et al. Complete genomes of Asgard archaea reveal diverse integrated and mobile genetic elements. *Genome Res* 2024;34:1595–609. 10.1101/gr.279480.124 39406503 PMC11529989

[26] Krupovic M, Makarova KS, Wolf YI et al. Integrated mobile genetic elements in Thaumarchaeota. *Environ Microbiol* 2019;21:2056–78. 10.1111/1462-2920.14564 30773816 PMC6563490

[27] Li R, Wang YM, Hu H et al. Metagenomic analysis reveals unexplored diversity of archaeal virome in the human gut. *Nat Commun* 2022;13:7978. 10.1038/s41467-022-35735-y 36581612 PMC9800368

[28] Nayfach S, Páez-Espino D, Call L et al. Metagenomic compendium of 189,680 DNA viruses from the human gut microbiome. *Nat Microbiol* 2021;6:960–70. 10.1038/s41564-021-00928-6 34168315 PMC8241571

[29] Camargo AP, Roux S, Schulz F et al. Identification of mobile genetic elements with geNomad. *Nat Biotechnol* 2024;42:1303–12. 10.1038/s41587-023-01953-y 37735266 PMC11324519

[30] Guo JR, Bolduc B, Zayed AA et al. VirSorter2: a multi-classifier, expert-guided approach to detect diverse DNA and RNA viruses. *Microbiome* 2021;9:37. 10.1186/s40168-020-00990-y 33522966 PMC7852108

[31] Martin C, Emerson JB, Roux S et al. A call for caution in the biological interpretation of viral auxiliary metabolic genes. *Nat Microbiol* 2025;10:2122–9. 10.1038/s41564-025-02095-4 40866482 PMC13284520

[32] Hampton HG, Watson BNJ, Fineran PC. The arms race between bacteria and their phage foes. *Nature* 2020;577:327–36. 10.1038/s41586-019-1894-8 31942051

[33] Ahlgren NA, Fuchsman CA, Rocap G et al. Discovery of several novel, widespread, and ecologically distinct marine Thaumarchaeota viruses that encode *amoC* nitrification genes. *ISME J* 2019;13:618–31. 10.1038/s41396-018-0289-4 30315316 PMC6462027

[34] Martínez-Alvarez L, Peng X. Diversity and evolution of archaeal immune strategies. *Nucleic Acids Res* 2026;54:gkag225. 10.1093/nar/gkag225 41830331 PMC12988326

[35] Tesson F, Huiting E, Wei LL et al. Exploring the diversity of anti-defense systems across prokaryotes, phages and mobile genetic elements. *Nucleic Acids Res* 2024;53:gkae1171. 10.1093/nar/gkae1171 PMC11724313 39657785

[36] Bhoobalan-Chitty Y, Xu SS, Martinez-Alvarez L et al. Regulatory sequence-based discovery of anti-defense genes in archaeal viruses. *Nat Commun* 2024;15:3699. 10.1038/s41467-024-48074-x 38698035 PMC11065993

[37] Medvedeva S, Borrel G, Krupovic M et al. A compendium of viruses from methanogenic archaea reveals their diversity and adaptations to the gut environment. *Nat Microbiol* 2023;8:2170–82. 10.1038/s41564-023-01485-w 37749252

[38] Zhang ZH, Liu YQ, Zhao WS et al. Distinct genes and microbial communities involved in nitrogen cycling between monsoon- and westerlies-dominated Tibetan glaciers. *Nat Commun* 2025;16:5926. 10.1038/s41467-025-61002-x 40595658 PMC12216452

[39] Liu YQ, Zhang ZH, Jiao NZ et al. Diverse novel RNA viruses in the cryosphere of the Tibetan plateau. *Natl Sci Rev* 2025;12:nwaf361. 10.1093/nsr/nwaf361 41190010 PMC12581897

[40] Zhao Y, Feng ML, Chi HF et al. Diversity, function andactivity of DNA viruses in the Qiangyong proglacial lake sediment, the Tibetan plateau. *Environ Microbiol Rep* 2026;18:e70262. 10.1111/1758-2229.70262 41508741 PMC12783922

[41] Nayfach S, Camargo AP, Schulz F et al. CheckV assesses the quality and completeness of metagenome-assembled viral genomes. *Nat Biotechnol* 2021;39:578–85. 10.1038/s41587-020-00774-7 33349699 PMC8116208

[42] Camacho C, Coulouris G, Avagyan V et al. BLAST plus: architecture and applications. *BMC Bioinformatics* 2009;10:421. 10.1186/1471-2105-10-421 20003500 PMC2803857

[43] Kieft K, Zhou ZC, Anantharaman K. VIBRANT: automated recovery, annotation and curation of microbial viruses, and evaluation of viral community function from genomic sequences. *Microbiome* 2020;8:90. 10.1186/s40168-020-00867-0 32522236 PMC7288430

[44] Shang JY, Tang XB, Sun YN. PhaTYP: predicting the lifestyle for bacteriophages using BERT. *Brief Bioinform* 2023;24:1–11. 10.1093/bib/bbac487 PMC9851330 36659812

[45] Bolduc B, Zablocki O, Turner D et al. Machine learning enables scalable and systematic hierarchical virus taxonomy. *Nat Biotechnol* 2025. 10.1038/s41587-025-02946-9 41420049

[46] Zheng KY, Sun JH, Liang YT et al. VITAP: a high precision tool for DNA and RNA viral classification based on meta-omic data. *Nat Commun* 2025;16:2226. 10.1038/s41467-025-57500-7 40044690 PMC11883027

[47] Jiang JZ, Yuan WG, Shang JY et al. Virus classification for viral genomic fragments using PhaGCN2. *Brief Bioinform* 2023;24:bbac505. 10.1093/bib/bbac505 36464489

[48] Camargo AP, Nayfach S, Chen IMA et al. IMG/VR v4: an expanded database of uncultivated virus genomes within a framework of extensive functional, taxonomic, and ecological metadata. *Nucleic Acids Res* 2023;51:D733–43. 10.1093/nar/gkac1037 36399502 PMC9825611

[49] Nishimura Y, Yoshida T, Kuronishi M et al. ViPTree: the viral proteomic tree server. *Bioinformatics* 2017;33:2379–80. 10.1093/bioinformatics/btx157 28379287

[50] Liu Y, Demina T, Roux S et al. Diversity, taxonomy, and evolution of archaeal viruses of the class *Caudoviricetes*. *PLoS Biol* 2021;19:e3001442. 10.1371/journal.pbio.3001442 34752450 PMC8651126

[51] Zhou YF, Zhou L, Yan SL et al. Diverse viruses of marine archaea discovered using metagenomics. *Environ Microbiol* 2023;25:367–82. 10.1111/1462-2920.16287 36385454

[52] Medvedeva S, Sun JR, Yutin N et al. Three families of Asgard archaeal viruses identified in metagenome-assembled genomes. *Nat Microbiol* 2022;7:962–73. 10.1038/s41564-022-01144-6 35760839 PMC11165672

[53] Rambo IM, Langwig MV, Leao P et al. Genomes of six viruses that infect Asgard archaea from deep-sea sediments. *Nat Microbiol* 2022;7:953–61. 10.1038/s41564-022-01150-8 35760837

[54] Tamarit D, Caceres EF, Krupovic M et al. A closed *Candidatus* Odinarchaeum chromosome exposes Asgard archaeal viruses. *Nat Microbiol* 2022;7:948–52. 10.1038/s41564-022-01122-y 35760836 PMC9246712

[55] Hyatt D, Chen GL, LoCascio PF et al. Prodigal: prokaryotic gene recognition and translation initiation site identification. *BMC Bioinformatics* 2010;11:119. 10.1186/1471-2105-11-119 20211023 PMC2848648

[56] Li WZ, Godzik A. Cd-hit: a fast program for clustering and comparing large sets of protein or nucleotide sequences. *Bioinformatics* 2006;22:1658–9. 10.1093/bioinformatics/btl158 16731699

[57] Couvin D, Bernheim A, Toffano-Nioche C et al. CRISPRCasFinder, an update of CRISRFinder, includes a portable version, enhanced performance and integrates search for Cas proteins. *Nucleic Acids Res* 2018;46:W246–51. 10.1093/nar/gky425 29790974 PMC6030898

[58] Russel J, Pinilla-Redondo R, Mayo-Muñoz D et al. CRISPRCasTyper: automated identification, annotation, and classification of CRISPR-Cas loci. *CRISPR J* 2020;3:462–9. 10.1089/crispr.2020.0059 33275853

[59] Tesson F, Hervé A, Mordret E et al. Systematic and quantitative view of the antiviral arsenal of prokaryotes. *Nat Commun* 2022;13:2561. 10.1038/s41467-022-30269-9 35538097 PMC9090908

[60] Néron B, Denise R, Coluzzi C et al. MacSyFinder v2: improved modelling and search engine to identify molecular systems in genomes. *Peer Community J* 2023;3:e28. 10.24072/pcjournal.250

[61] Roux S, Camargo AP, Coutinho FH et al. iPHoP: an integrated machine learning framework to maximize host prediction for metagenome-derived viruses of archaea and bacteria. *PLoS Biol* 2023;21:e3002083. 10.1371/journal.pbio.3002083 37083735 PMC10155999

[62] Yan YC, Zheng JF, Zhang XP et al. dbAPIS: a database of anti-prokaryotic immune system genes. *Nucleic Acids Res* 2023;52:D419–25. 10.1093/nar/gkad932 PMC10767833 37889074

[63] Buchfink B, Xie C, Huson DH. Fast and sensitive protein alignment using DIAMOND. *Nat Methods* 2015;12:59–60. 10.1038/nmeth.3176 25402007

[64] Eddy SR . Accelerated profile HMM searches. *PLoS Comput Biol* 2011;7:e1002195. 10.1371/journal.pcbi.1002195 22039361 PMC3197634

[65] Jones P, Binns D, Chang HY et al. InterProScan 5: genome-scale protein function classification. *Bioinformatics* 2014;30:1236–40. 10.1093/bioinformatics/btu031 24451626 PMC3998142

[66] Steinegger M, Meier M, Mirdita M et al. HH-suite3 for fast remote homology detection and deep protein annotation. *BMC Bioinformatics* 2019;20:473. 10.1186/s12859-019-3019-7 31521110 PMC6744700

[67] Shaffer M, Borton MA, McGivern BB et al. DRAM for distilling microbial metabolism to automate the curation of microbiome function. *Nucleic Acids Res* 2020;48:8883–900. 10.1093/nar/gkaa621 32766782 PMC7498326

[68] Luo XQ, Wang PD, Li JL et al. Viral community-wide auxiliary metabolic genes differ by lifestyles, habitats, and hosts. *Microbiome* 2022;10:190. 10.1186/s40168-022-01384-y 36333738 PMC9636769

[69] ter Horst AM, Santos-Medellín C, Sorensen JW et al. Minnesota peat viromes reveal terrestrial and aquatic niche partitioning for local and global viral populations. *Microbiome* 2021;9:233. 10.1186/s40168-021-01156-0 34836550 PMC8626947

[70] Minh BQ, Schmidt HA, Chernomor O et al. IQ-TREE 2: new models and efficient methods for phylogenetic inference in the genomic era. *Mol Biol Evol* 2020;37:1530–4. 10.1093/molbev/msaa015 32011700 PMC7182206

[71] Katoh K, Standley DM. MAFFT multiple sequence alignment software version 7: improvements in performance and usability. *Mol Biol Evol* 2013;30:772–80. 10.1093/molbev/mst010 23329690 PMC3603318

[72] Capella-Gutiérrez S, Silla-Martínez JM, Gabaldón T. trimAl: a tool for automated alignment trimming in large-scale phylogenetic analyses. *Bioinformatics* 2009;25:1972–3. 10.1093/bioinformatics/btp348 19505945 PMC2712344

[73] Letunic I, Bork P. Interactive tree of life (iTOL): an online tool for phylogenetic tree display and annotation. *Bioinformatics* 2007;23:127–8. 10.1093/bioinformatics/btl529 17050570

[74] Sheppard C, Werner F. Structure and mechanisms of viral transcription factors in archaea. *Extremophiles* 2017;21:829–38. 10.1007/s00792-017-0951-1 28681113 PMC5569661

[75] Mayo-Munoz D, Pinilla-Redondo R, Camara-Wilpert S et al. Inhibitors of bacterial immune systems: discovery, mechanisms and applications. *Nat Rev Genet* 2024;25:237–54. 10.1038/s41576-023-00676-9 38291236

[76] Flodman K, Tsai R, Xu MY et al. Type II restriction of bacteriophage DNA with 5hmdU-derived base modifications. *Front Microbiol* 2019;10:584. 10.3389/fmicb.2019.00584 30984133 PMC6449724

[77] Zhang ZQ, Liu Y, Wang S et al. Temperate membrane-containing halophilic archaeal virus SNJ1 has a circular dsDNA genome identical to that of plasmid pHH205. *Virology* 2012;434:233–41. 10.1016/j.virol.2012.05.036 22784791

[78] Mochizuki T, Sako Y, Prangishvili D. Provirus induction in hyperthermophilic archaea: characterization of *Aeropyrum pernix* spindle-shaped virus 1 and *Aeropyrum pernix* ovoid virus 1. *J Bacteriol* 2011;193:5412–9. 10.1128/jb.05101-11 21784945 PMC3187419

[79] Chen Z, Liu Y, Wang YX et al. A virus-borne DNA damage signaling pathway controls the lysogeny-induction switch in a group of temperate pleolipoviruses. *Nucleic Acids Res* 2023;51:3270–87. 10.1093/nar/gkad125 36864746 PMC10123103

[80] Zhong ZP, Du JJ, Koestlbacher S et al. Viral potential to modulate microbial methane metabolism varies by habitat. *Nat Commun* 2024;15:1857. 10.1038/s41467-024-46109-x 38424049 PMC10904782

[81] Pratama AA, Pérez-Carrascal O, Sullivan MB et al. Diversity and ecological roles of hidden viral players in groundwater microbiomes. *Nat Commun* 2026;17:2179. 10.1038/s41467-026-68914-2 41617723 PMC12960796

[82] Zhao Y, Zhang Z, Feng M et al. Functional and evolutionary characterization of potential auxiliary metabolic genes of the global RNA virome. *IMETAOMICS* 2025;2:e70002. 10.1002/imo2.70002 41675156 PMC12806420

[83] Roux S, Adriaenssens EM, Dutilh BE et al. Minimum information about an uncultivated virus genome (MIUViG). *Nat Biotechnol* 2019;37:29–37. 10.1038/nbt.4306 30556814 PMC6871006

[84] Graham EB, Camargo AP, Wu RN et al. A global atlas of soil viruses reveals unexplored biodiversity and potential biogeochemical impacts. *Nat Microbiol* 2024;9:1873–83. 10.1038/s41564-024-01686-x 38902374 PMC11222151

