# Supplementary material for: Unraveling the diversity, function, and virus–host interactions of archaeal proviruses
Source: ISME Commun. 2026 Aug 22;6(1):ycag243. doi: 10.1093/ismeco/ycag243 (PMC13573593; doi:10.1093/ismeco/ycag243)
Supplement: Supplementary file 1 [file supplementary_material_ycag243.zip › Archaea_virus_Supporting_information-0820_ycag243.docx]

**Supporting information to：**

**Unraveling the diversity, function, and virus-host interactions of archaeal proviruses**

Yang Zhao^1,2,3,4#^, PengFei Liu^1,2,3#*^, LinXing Chen^5*^, MeiLing Feng^1,2,3,4^, Rong Wen^1,2,3,4^, ZhiHao Zhang^1,2,3^, XueFeng Zhang^6,7^, HongFei Chi^1,2,3^

^1^Center for Pan-third Pole Environment, Lanzhou University, Lanzhou, China

^2^Key Laboratory of Pan-third Pole Biogeochemical Cycling, Lanzhou, China

^3^Chayu Monsoon Corridor Observation and Research Station for Multi-Sphere Changes, Chayu, China

^4^College of Ecology, Lanzhou University, Lanzhou, China

^5^State Key Laboratory of Advanced Environmental Technology, School of Environment, University of Science and Technology of China, Hefei, China

^6^State Key Laboratory of Tibetan Plateau Earth System, Environment and Resources (TPESER), Institute of Tibetan Plateau Research, Chinese Academy of Sciences, Beijing, China

^7^University of Chinese Academy of Sciences, Beijing, China

\# These authors contribute equally.

**The Supplementary Information contains 17 pages with 5 supplementary methods, 1 supplementary result, and 6 supplementary figures.**

**Supplementary Methods**

Method S1. Archaea genome collection

Method S2. Lifestyle prediction of proviruses

Method S3. Taxonomic classification of vOTUs

Method S4. Protein function annotation and auxiliary metabolic genes (AMGs) identification

Method S5. Statistical analyses and visualization

**Supplementary** **Result**

Result S1. Antiviral systems in archaea

**Supplementary Figures**

Figure S1. Global characteristics of archaeal proviruses and their hosts.

Figure S2. The proportion of archaeal provirus communities at the phylum, class, order, and family levels.

Figure S3. Maximum-likelihood phylogenetic tree of MCPs encoded by archaeal proviruses.

Figure S4. Genomic comparison of 16 groups of novel archaeal proviruses.

Figure S5. Maximum-likelihood phylogenetic tree of NarC homologs, including provirus AMGs and reference sequences.

Figure S6. Genetic architectures of CRISPR–Cas systems in archaea.

## Supplementary Methods

### Method S1. Archaea genome collection

This study collected a total of 25,530 archaeal genomes, comprising 24,840 genomes (including cultured isolates, metagenome-assembled genomes (MAGs), and single-amplified genomes (SAGs)) obtained from the NCBI GenBank database (November 2024), and 690 archaeal MAGs from the cryosphere environments of the Tibetan Plateau which have been published in our previous studies^1-3^. Following data collection, we performed quality screening and taxonomic validation on all archaeal genomes. Specifically, CheckM2 v1.0.12^4^ was used to evaluate genome completeness and contamination, and low-quality genomes (completeness < 50% and contamination > 10%) were excluded. The retained genomes were subsequently dereplicated using dRep v3.3.0^5^ with an Average Nucleotide Identity (ANI) cutoff value of 99.99%. This threshold was selected to remove only nearly identical genomes while preserving genomes that may contain distinct proviruses, thereby maximizing the recovery of archaeal provirus diversity. Taxonomic classification and validation were performed using GTDB-Tk v2.4.1 (reference data r226)^6^, confirming all as Archaea. Ultimately, our study generated a non-redundant set of 21,989 medium- to high-quality archaeal genomes (21,593 from the NCBI GenBank database and 396 from our own). These archaea were classified into 21 phyla, 65 classes, 172 orders, 603 families, and 2,075 genera. Among these, there were 48 families with more than 100 genomes, 33 families with 50 to 100 genomes, 25 families with 30 to 50 genomes, and the remaining 496 families with less than 30 genomes (see Table S1 for details).

### Method S2. Lifestyle prediction of proviruses

The lifestyle of proviruses was predicted using four methods. (i) CheckV v1.0.1 predicts lysogenic lifestyle by detecting provirus boundaries^7^. (ii) The proviruses with proteins annotated (see below, DRAMv and HH-suite3 annotation of viral proteins) as integrases, invertase, serine recombinases, transposase, *parA/B*, and CI/Cro repressor are considered proviruses^8^. CheckV and gene annotation were used only as evidence supporting a lysogenic lifestyle and were not used to infer a lytic lifestyle. (iii) PhaTYP v0.3.0 (minimum score ≥ 0.9)^9^ and iv) VIBRANT v1.2.1^10^ with default parameters were subsequently used to further distinguish lysogenic and lytic lifestyles. However, it should be noted that PhaTYP employs a BERT-based deep learning framework to predict viral lifestyles based on protein sequence features^9^. The lifestyle prediction model was trained primarily on bacteriophage datasets rather than archaeal viruses, which may introduce prediction bias when applied to archaeal viruses. To address potential biases from PhaTYP’s bacteriophage training data, we additionally used VIBRANT, which leverages curated viral protein families (including those from archaeal viruses) and a machine learning framework^10^. The combined result of more than two software was used as the final prediction result. Conflicting results between two software were resolved through sequential priority assignment to CheckV, gene annotation, VIBRANT, and PhaTYP outputs. Proviruses that could not be assigned a lifestyle by any of the four approaches were classified as having an unknown lifestyle.

### Method S3. Taxonomic classification of vOTUs

The obtained vOTUs were taxonomically classified using geNomad v1.11.0^11^, vConTACT3 v3.1.6^12^, VITAP v1.7.1^13^, PhaGCN3 v3.1^14^, PhaGCN2 v2.3^15^, and viral genome homolog search method based on BLASTn comparison of the IMG/VR V4 database^16^. Briefly, geNomad with the default parameter was used to classify vOTU by taxonomic rank of annotated proteins. For vConTACT3, vOTUs were classified using default parameters with the "NCBI Viruses RefSeq v.230" database. VITAP was employed with default parameters, applying a lineage score cutoff of ≥ 0.9 for taxonomic classification. PhaGCN3 and PhaGCN2 classified vOTUs at the family-level using recommended cutoff scores > 0.5^15^. For the BLASTn-based approach, taxonomy was assigned based on the top best hit in the IMG/VR V4 database that met the thresholds of ≥ 95% ANI and ≥ 85% AF^17^. The final taxonomic assignment for each vOTU was determined through hierarchical integration of results from the six pipelines, prioritized in the following order: BLASTn, vConTACT3, geNomad, VITAP, PhaGCN3, and PhaGCN2. If conflicts existed at a lower taxonomic level (e.g., family level) for different pipelines but there was a common higher level of taxonomy (e.g., order level), then the higher taxonomic level was assigned.

To further clarify the family-level taxonomic affiliation of unclassified complete proviruses encoding major capsid proteins (MCPs) belonging to *Caudoviricetes*, a proteomic tree was constructed using ViPTree web server v4.0^18^ with default parameters. The input dataset comprised three components: (i) 21 complete archaeal proviruses encoding MCPs identified in this study; (ii) recently reported archaeal viral families within the class *Caudoviricetes*, including *Zhurongvirus*, *Houtuvirus*, *Nuwavirus*, *Houyivirus*, *Gonggongvirus*, *Danacaviridae*, *Dallocaviridae*, *Assalcaviridae*, *Quasboviridae*, *Gulliviridae*, *Lilliviridae*, and *Blefusviridae*^19, 20^; and (iii) reference archaeal viral genomes belonging to *Caudoviricetes* obtained from the latest version of ICTV Virus Metadata Resource (VMR_MSL40_v2).

### Method S4. Protein function annotation and auxiliary metabolic genes (AMGs) identification

Protein-coding sequences of proviruses were predicted using Prodigal v2.6.3 (parameter: -p meta) ^21^, and the resulting protein sequences were annotated using InterProScan (v5.76-107.0)^22^ against CDD v3.21^23^, Pfam 38.0^24^, NCBIfam v17.0, SUPERFAMILY v1.75^25^, Gene3D v4.3.0^26^, PRINTS v.42.0^27^, Phobius v.1.0.1^28^, MobiDBLite v.2.0^29^, and TMHMM v.2.0c^30^ database. For proteins with unknown functions, multiple sequence alignment of each protein was generated using HHblits v3.3.0^31^ with UniRef30_2023_02 database (3 MSA generation iterations; e-value, 1e-6), and then searched against a series of publicly available databases such as PDB70_May_2025^32^, PHROG v4^33^, SCOPe70 v2.08^34^ and UniProt-SwissProt-viral70_Nov_2021^35^ using HHsearch v3.3.0 (parameter: -Z 250 -loc -z 1 -b 1 -B 250 -ssm 2 -sc 1 -seq 1 -norealign -maxres 32000).

AMG prediction was performed using the DRAM-v pipeline^36^ for the 537 proviruses encoding at least one viral hallmark gene, including MCP, terminase large subunit (TerL), or portal proteins. Briefly, these proviruses were re-analyzed using VirSorter2 (parameters: -prep-for-dramv -min-length 5000 -min-score 0 -include-groups dsDNAphage,ssDNA,NCLDV,RNA,lavidaviridae) to generate an “affi-contigs.tab” file, which was subsequently annotated using DRAM-v with the default database. The “-min-score 0” parameter was employed to ensure maximal sensitivity, as some proviruses derived from geNomad were not classified as viral contigs by VirSorter2 when using the “-min-score 0.5” parameter , as previously described^37^. Predicted AMGs were filtered based on the following criteria: auxiliary_score ≤ 3 and amg_flag assigned to M or M with E and/or K. Additionally, the flanking genes of the putative AMG were manually inspected to ensure the presence of viral marker genes or viral-like genes.

To provide insight into the potential functional relevance of AMG to the host, we first compared provirus sequences to the host genome using BLASTn (parameters: -perc_identity 100 -qcov_hsp_perc 100 -evalue 1e-3) to determine that the host genome was unintegrated or uncontaminated with viral sequences. Subsequently, the AMG was compared to the host genome using BLASTp to assess the relatedness of the identified AMG to homologous genes found in the host^38^. The three-dimensional structures of AMGs were predicted using both AlphaFold3^39^ and Phyre^2^ pipeline (http://www.sbg.bio.ic.ac.uk/phyre2)^40^.

### Method S5. Statistical analyses and visualization

All statistical analyses in this study were performed using R v4.1.4. Mann–Whitney U tests were performed using the wilcox.test function in the R “stats” package to evaluate differences in host and provirus GC content across phyla, as well as to compare genome size and G+C content between archaeal genomes with and without proviruses. The manuscript figures were mainly generated using the R packages “ggplot2”, “ggpubr”, “dplyr”, “igraph” and “gggene”. The figure format was adjusted using Adobe Illustrator if needed.

### Supplementary Result

### Result S1. Antiviral systems in archaea

A total of 1,297 complete CRISPR-Cas systems were identified in 4.9% (1,070/21,989) of archaeal genomes analyzed, and these CRISPR-Cas systems can be categorized into 2 classes, 5 types, and 29 subtypes (Figure S6; Table S12). Specifically, class 1 CRISPR-Cas systems, encompassing types I, III, and IV, were identified in 1,045 archaeal genomes spanning 14 phyla, with Halobacteriota (*n* = 527; 13 subtypes), Thermoproteota (*n* = 380; 18 subtypes), Methanobacteriota (*n* = 136; 7 subtypes), and Methanobacteriota_B (*n* = 108; 8 subtypes) exhibiting the highest number and subtype diversity. In contrast, class 2 CRISPR-Cas systems (types II, V) were found in only 25 genomes across four phyla, including Thermoplasmatota (*n* = 14; subtype II-A or V-A), Nanobdellota (*n* = 7; V-A or V-F1), Micrarchaeota (*n* = 2; II-C2 or V-F1), and Halobacteriota (*n* = 2; II-A) (Figure S6; Table S12).

Besides CRISPR-Cas, archaea have evolved other antiviral arsenals during their arms race with viruses. A total of 35,299 non-CRISPR-Cas antiviral systems were identified across 51.5% (*n* = 11,328) of archaeal genomes, spanning 177 distinct defense system families. Among these, the restriction-modification (RM) system family is the most prevalent, present in 35.0% of genomes, followed by Prometheus (8.3%), SoFIC (6.7%), and Ceres (5.6%) (Table S13). Notably, 160 system families were detected in fewer than 1% of the genomes. Some systems (e.g., Shedu), while less abundant, are widely distributed across most phyla. In contrast, some other systems are confined to specific phyla, such as Toutatis, Erebus, and Brigantia, which are exclusively restricted to Thermoproteota. Strikingly, 85 of all identified antiviral systems in archaea are also encoded by proviruses, including RM, HEC-05, BREX, and CRISPR-Cas (Table S14).

# References

1. Zhang Z H, Liu Y Q, Zhao W S, Liu K S, Chen Y Y, Wang F, et al. Distinct genes and microbial communities involved in nitrogen cycling between monsoon- and westerlies-dominated Tibetan glaciers. *Nature Communications*. 2025; 16:5926. <https://doi.org/10.1038/s41467-025-61002-x>

2. Liu Y Q, Zhang Z H, Jiao N Z, Zhu Y G, Zhang R, Dominguez-Huerta G, et al. Diverse novel RNA viruses in the cryosphere of the Tibetan Plateau. *National Science Review*. 2025; 12:nwaf361. <https://doi.org/10.1093/nsr/nwaf361>

3. Zhao Y, Feng M L, Chi H F, Liu K S, Wen R, Zhang W Z, et al. Diversity, function andactivity of DNA viruses in the Qiangyong proglacial lake sediment, the Tibetan Plateau. *Environmental Microbiology Reports*. 2026; 18:e70262. <https://doi.org/10.1111/1758-2229.70262>

4. Parks D H, Imelfort M, Skennerton C T, Hugenholtz P, Tyson G W. CheckM: assessing the quality of microbial genomes recovered from isolates, single cells, and metagenomes. *Genome Research*. 2015; 25:1043-1055. <https://doi.org/10.1101/gr.186072.114>

5. Olm M R, Brown C T, Brooks B, Banfield J F. dRep: a tool for fast and accurate genomic comparisons that enables improved genome recovery from metagenomes through de-replication. *ISME Journal*. 2017; 11:2864-2868. <https://doi.org/10.1038/ismej.2017.126>

6. Chaumeil P A, Mussig A J, Hugenholtz P, Parks D H. GTDB-Tk v2: memory friendly classification with the genome taxonomy database. *Bioinformatics*. 2022; 38:5315-5316. <https://doi.org/10.1093/bioinformatics/btac672>

7. Nayfach S, Camargo A P, Schulz F, Eloe-Fadrosh E, Roux S, Kyrpides N C. CheckV assesses the quality and completeness of metagenome-assembled viral genomes. *Nature Biotechnology*. 2021; 39:578-585. <https://doi.org/10.1038/s41587-020-00774-7>

8. Jian H H, Yi Y, Wang J H, Hao Y L, Zhang M J, Wang S Y, et al. Diversity and distribution of viruses inhabiting the deepest ocean on Earth. *ISME J.* 2021; 15:3094-3110. <https://doi.org/10.1038/s41396-021-00994-y>

9. Shang J Y, Tang X B, Sun Y N. PhaTYP: predicting the lifestyle for bacteriophages using BERT. *Briefings in Bioinformatics*. 2023; 24:1–11. <https://doi.org/10.1093/bib/bbac487>

10. Kieft K, Zhou Z C, Anantharaman K. VIBRANT: automated recovery, annotation and curation of microbial viruses, and evaluation of viral community function from genomic sequences. *Microbiome*. 2020; 8:90. <https://doi.org/10.1186/s40168-020-00867-0>

11. Camargo A P, Roux S, Schulz F, Babinski M, Xu Y, Hu B, et al. Identification of mobile genetic elements with geNomad. *Nature Biotechnology*. 2024; 42:1303–1312. <https://doi.org/10.1038/s41587-023-01953-y>

12. Bolduc B, Zablocki O, Turner D, Bin Jang H, Guo J R, Adriaenssens E M, et al. Machine learning enables scalable and systematic hierarchical virus taxonomy. *Nature Biotechnology*. 2025; <https://doi.org/10.1038/s41587-025-02946-9>

13. Zheng K Y, Sun J H, Liang Y T, Kong L L, Paez-Espino D, McMinn A, et al. VITAP: a high precision tool for DNA and RNA viral classification based on meta-omic data. *Nature Communications*. 2025; 16:2226. <https://doi.org/10.1038/s41467-025-57500-7>

14. Xia H L, Liang P Y, Yuan W G, Cao X D, Sun Y N, Jiang J Z, et al. PhaGCN_Cluster: A Scalable and Robust Framework for Automated Classification and Discovery of Viral Dark Matter from Metagenomes. *Interdisciplinary Sciences-Computational Life Sciences*. 2026; <https://doi.org/10.1007/s12539-026-00820-z>

15. Jiang J Z, Yuan W G, Shang J Y, Shi Y H, Yang L L, Liu M, et al. Virus classification for viral genomic fragments using PhaGCN2. *Brief. Bioinform.* 2023; 24:bbac505. <https://doi.org/10.1093/bib/bbac505>

16. Camargo A P, Nayfach S, Chen I M A, Palaniappan K, Ratner A, Chu K, et al. IMG/VR v4: an expanded database of uncultivated virus genomes within a framework of extensive functional, taxonomic, and ecological metadata. *Nucleic Acids Research*. 2023; 51:D733-D743. <https://doi.org/10.1093/nar/gkac1037>

17. Jarett J K, Dzunková M, Schulz F, Roux S, Paez-Espino D, Eloe-Fadrosh E, et al. Insights into the dynamics between viruses and their hosts in a hot spring microbial mat. *ISME Journal*. 2020; 14:2527-2541. <https://doi.org/10.1038/s41396-020-0705-4>

18. Nishimura Y, Yoshida T, Kuronishi M, Uehara H, Ogata H, Goto S. ViPTree: the viral proteomic tree server. *Bioinformatics*. 2017; 33:2379-2380. <https://doi.org/10.1093/bioinformatics/btx157>

19. Zhou Y F, Gutiérrez-Preciado A, Liu Y, Moreira D, Yakimov M M, López-García P, et al. Viruses and virus satellites of haloarchaea and their nanosized DPANN symbionts reveal intricate nested interactions. *Nature Microbiology*. 2025; 10:2673–2685. <https://doi.org/10.1038/s41564-025-02149-7>

20. Wu Z Z, Liu S F, Ni J R. Metagenomic characterization of viruses and mobile genetic elements associated with the DPANN archaeal superphylum. *Nature Microbiology*. 2024; 9:3362–3375. <https://doi.org/10.1038/s41564-024-01839-y>

21. Hyatt D, Chen G L, LoCascio P F, Land M L, Larimer F W, Hauser L J. Prodigal: prokaryotic gene recognition and translation initiation site identification. *BMC Bioinformatics*. 2010; 11:119. <https://doi.org/10.1186/1471-2105-11-119>

22. Jones P, Binns D, Chang H Y, Fraser M, Li W Z, McAnulla C, et al. InterProScan 5: genome-scale protein function classification. *Bioinformatics*. 2014; 30:1236-1240. <https://doi.org/10.1093/bioinformatics/btu031>

23. Wang J Y, Chitsaz F, Derbyshire M K, Gonzales N R, Gwadz M, Lu S N, et al. The conserved domain database in 2023. *Nucleic Acids Research*. 2023; 51:D384-D388. <https://doi.org/10.1093/nar/gkac1096>

24. Mistry J, Chuguransky S, Williams L, Qureshi M, Salazar G A, Sonnhammer E L L, et al. Pfam: The protein families database in 2021. *Nucleic Acids Research*. 2021; 49:D412-D419. <https://doi.org/10.1093/nar/gkaa913>

25. Wilson D, Madera M, Vogel C, Chothia C, Gough J. The SUPERFAMILY database in 2007: families and functions. *Nucleic Acids Research*. 2007; 35:D308-D313. <https://doi.org/10.1093/nar/gkl910>

26. Lewis T E, Sillitoe I, Dawson N, Lam S D, Clarke T, Lee D, et al. Gene3D: Extensive prediction of globular domains in proteins. *Nucleic Acids Research*. 2018; 46:D435-D439. <https://doi.org/10.1093/nar/gkx1069>

27. Attwood T K, Coletta A, Muirhead G, Pavlopoulou A, Philippou P B, Popov I, et al. The PRINTS database: a fine-grained protein sequence annotation and analysis resource-its status in 2012. *Database-the Journal of Biological Databases and Curation*. 2012; 2012:bas019. <https://doi.org/10.1093/database/bas019>

28. Käll L, Krogh A, Sonnhammer E L L. Advantages of combined transmembrane topology and signal peptide prediction -: the Phobius web server. *Nucleic Acids Research*. 2007; 35:W429-W432. <https://doi.org/10.1093/nar/gkm256>

29. Potenza E, Di Domenico T, Walsh I, Tosatto S C E. MobiDB 2.0: an improved database of intrinsically disordered and mobile proteins. *Nucleic Acids Research*. 2015; 43:D315-D320. <https://doi.org/10.1093/nar/gku982>

30. Krogh A, Larsson B, von Heijne G, Sonnhammer E L L. Predicting transmembrane protein topology with a hidden Markov model: Application to complete genomes. *Journal of Molecular Biology*. 2001; 305:567-580. <https://doi.org/10.1006/jmbi.2000.4315>

31. Steinegger M, Meier M, Mirdita M, Vöhringer H, Haunsberger S J, Söding J. HH-suite3 for fast remote homology detection and deep protein annotation. *BMC Bioinformatics*. 2019; 20:473. <https://doi.org/10.1186/s12859-019-3019-7>

32. Berman H M, Westbrook J, Feng Z, Gilliland G, Bhat T N, Weissig H, et al. The Protein Data Bank. *Nucleic Acids Research*. 2000; 28:235-242. <https://doi.org/10.1093/nar/28.1.235>

33. Terzian P, Ndela E O, Galiez C, Lossouarn J, Bucio R E P, Mom R, et al. PHROG: families of prokaryotic virus proteins clustered using remote homology. *Nar Genomics and Bioinformatics*. 2021; 3:lqab067. <https://doi.org/10.1093/nargab/lqab067>

34. Chandonia J M, Guan L, Lin S Y, Yu C H, Fox N K, Brenner S E. SCOPe: improvements to the structural classification of proteins - extended database to facilitate variant interpretation and machine learning. *Nucleic Acids Research*. 2022; 50:D553-D559. <https://doi.org/10.1093/nar/gkab1054>

35. Bateman A, Martin M J, Orchard S, Magrane M, Ahmad S, Alpi E, et al. UniProt: the Universal Protein Knowledgebase in 2023. *Nucleic Acids Research*. 2023; 51:D523-D531. <https://doi.org/10.1093/nar/gkac1052>

36. Shaffer M, Borton M A, McGivern B B, Zayed A A, La Rosa S L, Solden L M, et al. DRAM for distilling microbial metabolism to automate the curation of microbiome function. *Nucleic Acids Res.* 2020; 48:8883-8900. <https://doi.org/10.1093/nar/gkaa621>

37. Liu Y Q, Jiao N Z, Zhong K X, Zang L, Zhang R, Xiao X, et al. Diversity and function of mountain and polar supraglacial DNA viruses. *Sci. Bull.* 2023; 68:2418-2433. <https://doi.org/10.1016/j.scib.2023.09.007>

38. Gios E, Mosley O E, Hoggard M, Handley K M. High niche specificity and host genetic diversity of groundwater viruses. *ISME J.* 2024; 18:wrae035. <https://doi.org/10.1093/ismejo/wrae035>

39. Abramson J, Adler J, Dunger J, Evans R, Green T, Pritzel A, et al. Accurate structure prediction of biomolecular interactions with AlphaFold 3. *Nature*. 2024; 630:<https://doi.org/10.1038/s41586-024-07487-w>

40. Kelley L A, Mezulis S, Yates C M, Wass M N, Sternberg M J E. The Phyre2 web portal for protein modeling, prediction and analysis. *Nature Protocols*. 2015; 10:845-858. <https://doi.org/10.1038/nprot.2015.053>

**Supplementary figures**

**Figure S1. Global characteristics of archaeal proviruses and their hosts.** (a) The number of provirus regions carried by each strain. (b) The top, middle, and bottom rows of the Sankey diagram show the provirus length, CheckV quality, and CheckV completeness, respectively. (c) Comparative analyses of archaeal provirus and host genome sizes among the three archaeal phyla containing more than 100 proviruses. (d) Comparison of GC content between proviruses and their archaeal host genomes across different host phyla. Statistical significance in panels (c) and (d) was assessed using the Wilcoxon rank-sum test (***, *p* < 0.001).

**Figure S2. The proportion of archaeal provirus communities at the phylum, class, order, and family levels.**

**Figure S3. Maximum-likelihood phylogenetic tree of MCPs encoded by archaeal proviruses.** Complete archaeal provirus genomes are indicated by red font. Reference viruses are shown in black font, with their hosts in brackets. AlphaFold3-predicted MCP structural models are displayed around the tree.

**Figure S4. Genomic comparison of 16 groups of novel archaeal proviruses.** Homologous genes are displayed using the same colors, and the percentage of sequence identity at the protein level is indicated by different shades of grey. Annotated genes related to viral hallmarks are denoted by the arrows with red border.

**Figure S5. Maximum-likelihood phylogenetic tree of NarC homologs, including provirus AMGs and reference sequences**.

**Figure S6. Genetic architectures of CRISPR–Cas systems in archaea.** Genetic architectures of each subtype of the identifiable CRISPR–Cas system in archaeal phyla. If one subtype is possessed by multiple genomes for an individual phylum, the representative one is shown. CRISPR–Cas system subtypes belonging to classes 1 and 2 are in red and blue text, respectively, on the top right corner within each frame.

**Figure S6. Continue.**

**Figure S6. Continue.**
